# Climate, Rotation, and Tillage Impacts on Soybean Yield Gains in a 50‐Year Experiment

**DOI:** 10.1111/gcb.70469

**Published:** 2025-09-08

**Authors:** Raziel A. Ordóñez, Shaun N. Casteel, Rachel H. Stevens, Sotirios V. Archontoulis, Tony J. Vyn

**Affiliations:** ^1^ Department of Agronomy Purdue University West Lafayette Indiana USA; ^2^ Department of Agronomy Iowa State University Ames Iowa USA

**Keywords:** climate changes, crop rotation, long‐term experiment, soybean, tillage, yield gain

## Abstract

Understanding how interactive management practices and climatic behavior influence soybean [ *Glycine max* (L.) Merr.] productivity is imperative to inform future production systems under changing climate. This study examined eight unique long‐term production systems from 1975 to 2024 on a fertile rainfed Mollisol in Indiana, USA. Using this 400‐unit field‐scale dataset comparing soybean in monocropping versus following maize ( *Zea mays* L.) across four tillage intensities (Moldboard Plow, Chisel, Ridge/Strip‐Till and No‐Till), we investigated how these practices interact with each other and with weather patterns. Our focus was on the spring (1 April–20 June) and summer (21 June–30 September) periods, and their effects on final yields and morphometric plant phenes. Soybean yield results during the 50‐year period, when spring temperatures increased by 1.6°C, reflected (i) consistently positive yield responses to higher spring temperatures, at the rate of 796 kg ha^−1^/°C, especially in monocropping, despite concurrent development of wetter springs and drier summers; (ii) improved yield resilience in the No‐Till versus tilled systems; (iii) average soybean yields rotated with maize were 7.7% above those in monocropping but varied widely with tillage and year; and (iv) yield gain rates over time averaging 29.5 kg ha^−1^ year^−1^ for monocropping and 25.6 kg ha^−1^ year^−1^ for rotation. Plant height at 4‐ and 8‐weeks post‐planting was more influenced by air temperatures than precipitation, but final yield differences among tillage intensities were proportionately much smaller than relative soybean height differences in spring. These findings of consistent yield gains across multiple rotation and tillage regimes, despite changing climate factors, can inform actionable strategies for sustainable food production in future warming climate scenarios. Additionally, the unique rotation/tillage outcomes for 50 years provide a unique baseline for process‐based crop model calibration to enhance our ability to design future cropping systems.

## Graphical abstract

Soybean productivity increases from 1975 to 2024 in a rainfed Mollisol experiment in Indiana, USA, were shaped by genetic improvements, crop rotation, tillage, and climate. Despite rising spring temperatures (+1.6°C), yields increased steadily even in monocrop soybean and showed greater resilience in No‐Till systems. Annual yield gains averaging 29.5 kg ha^−1^ year^−1^ in continuous soybean and 25.6 kg ha^−1^ year^−1^ in rotation with maize varied among four tillage systems. Yields were more responsive to variation in spring warming than to precipitation. These 50‐year findings offer rare long‐term insights into crop‐climate‐management interactions and provide empirical benchmarks for biophysical model calibration and future climate‐adapting soybean production scenarios.

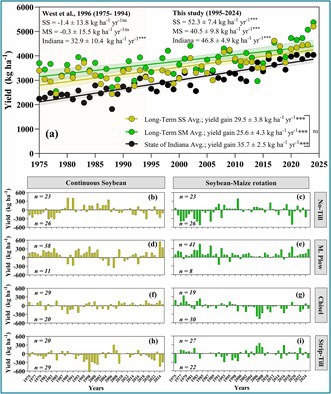

## 1 Introduction

Understanding how soybean yields respond to management practices such as rotation and tillage within changing weather patterns is imperative for assessing cropping system dynamics and improving climate change adaptation strategies (Howden et al. 2007; Battisti and Naylor 2009; Ray et al. 2012). Soybean is a critical global commodity crop; it holds significant importance in providing essential proteins, vegetable oils for human diets and animal feed, and biofuels (Rotundo et al. 2016; Assefa et al. 2019; Moro Rosso et al. 2021). However, increases in air temperatures are likely to reduce soybean yields, with losses further exacerbated by precipitation shortages during critical developmental stages (Lobell and Field 2007; Hoffman et al. 2020; Severini et al. 2024). Long‐term field experiments have played a pivotal role in advancing agricultural science (Aref and Wander 1997; Robertson et al. 2008) and have clarified the impacts of agricultural practices on crop system performance (Johnston and Poulton 2018). Yet, the long‐term impacts of crop rotation and tillage methods on soybean [ *Glycine max* (L.) Merr.] yields under changing climate conditions, and their roles in fostering a resilient agricultural system, are not well understood. Therefore, enhancing our understanding of these traditional agricultural practices is imperative, particularly in the context of adopting improved crop varieties as technology and weather shifts occur (Tester and Langridge 2010).

Global soybean production has steadily increased to 378 million metric tons, with Brazil leading at 40.7%, followed by the USA (29.9%), Argentina (13.2%) and China (5.3%); global production is projected to continue growing through 2050 (Bao et al. 2015; USDA‐FAS 2023). However, the rapid expansion of soybean cultivation, particularly in South America, poses significant risks (Gollnow et al. 2018; Song et al. 2021; Costantini and Bacenetti 2021), including soil carbon depletion through the conversion of carbon‐rich forest into cropland (Aragão et al. 2022), as well as biodiversity loss and damage to ecosystem integrity (Phalan et al. 2011; Grantham et al. 2020). Soybean cultivation spreading into newer production regions, such as Europe, highlights the crop's potential in areas with distinct climatic and soil conditions (Nendel et al. 2022; Rotundo et al. 2024). Yields in rainfed agroecosystems from the UK through southern Europe average 3.1 Mg ha^−1^ (Mazzoncini et al. 2008; Zimmer et al. 2016; Coleman et al. 2021; Karges et al. 2022; Döttinger et al. 2023; Simon‐Miquel et al. 2024), which compares favorably with the 3.5 Mg ha^−1^ yield average reported for North America cropping areas (USDA‐FAS 2023; Rotundo et al. 2024). Further expansion of soybean acreage amplifies the urgent need for identifying management practices that mitigate both land degradation and boost productivity on existing farmlands.

Research has demonstrated numerous benefits of both short‐ and long‐term soybean rotations (Seifert et al. 2017; Zhou et al. 2024). There is general recognition that any crop production system beyond monocropping may enhance soil physiochemical properties, nutrient cycling, and pest management, ultimately reducing reliance on chemical inputs and boosting crop yields (Liebman and Dyck 1993; Agomoh et al. 2021; Bybee‐Finley et al. 2024). In North America (USA and Canada), soybean production commonly occurs in short rotations of 2‐ and 3‐year cycles with maize ( *Zea mays* L.) and/or wheat ( *Triticum aestivum* L.) to achieve economic (i.e., stable yields and lower inputs) and soil health benefits such as global warming mitigation, habitat for soil microbes, and greater biodiversity (McDaniel et al. 2014; Bowles et al. 2020; Yang et al. 2024). Extended rotations incorporating crop species diversity beyond maize have shown even greater yield benefits (Janovicek et al. 2021; Agomoh et al. 2021; Costa et al. 2024). However, rotation‐derived yield benefits are not always clear and sometimes appear contradictory. Some older long‐term studies have reported soybean yield benefits of 8% to 25% when following maize (Crookston et al. 1991; Lund et al. 1993; Pedersen and Lauer 2003). In contrast, an intermediate‐term study of 15 years found no significant differences between soybean‐maize rotation and soybean monocropping (Morrison et al. 2017), and even soybean‐maize rotation yield penalties of up to 10.3% in some cases (Seifert et al. 2017). A 28‐year study reported higher soybean yields in 67% of the experimental years when soybeans were rotated with maize, compared to monocropping (Sindelar et al. 2015). Prior rotation studies have rarely involved a true monocropping treatment where soybean was the sole crop grown for longer than 15 years. Even longer‐term rotational studies with more management system alternatives would greatly improve our knowledge and subsequent model‐based projections, in the context of global warming and a growing global population.

Alterations in soil properties resulting from tillage practices significantly influence soybean growth and productivity (Vyn and Raimbault 1993), leading to divergent effects on yields depending on the rotation systems, weather conditions, and soil types (Pittelkow et al. 2015; Morrison et al. 2017). No tillage (No‐Till) systems, considered fundamental for sustainable agriculture, offer substantial benefits to soil health, including improvements in organic carbon, cation‐exchange capacity, hydraulic conductivity, and water retention (Gál et al. 2007; Powlson et al. 2014; Nunes et al. 2018). Conversely, conventional tillage practices, while promoting faster early‐season growth, can degrade soil structure, accelerate erosion, and deplete organic matter, compromising long‐term soil fertility and nutrient cycles (Hill 1990; Lal 1991; Kladivko 2001; Kahlon et al. 2013). While Powlson et al. (2014) argued that the potential of No‐Till systems to mitigate climate change is limited and often overstated, this claim has yet to be tested in soybean crops within the context of long‐term studies. Limited evidence suggests that a No‐Till system combined with rotation can enhance adaptation of agriculture to climate change, and can contribute to sustainability and food security (Gál et al. 2007; Teng et al. 2024; Gautam et al. 2025), although the full potential and yield stability of No‐Till systems remain unknown.

Despite the established benefits of No‐Till in soybean‐maize rotations (Zhou et al. 2024), earlier studies have reported mixed results for soybean. For instance, Wilhelm and Wortmann (2004) reported yield benefits of 5.3% with Moldboard Plow, 5.1% with Chisel, and 13.2% in Strip‐Till compared to No‐Till over a 16‐year period for soybean rotated with maize. In contrast, Karlen et al. (1998) found smaller rotation yield benefits for soybean after maize of just 2.1% in Moldboard Plow, 1.5% in Chisel, and a decrease of 2.8% for Strip‐Till in a 15‐year study. In their 20‐year study on Chalmer silty clay loam soil, West et al. (1996) found that while the Moldboard Plow outperformed other treatments in terms of soybean yield and plant height (for both in monocropping and soybean after maize), no significant differences were observed among the other tillage methods.

Height has remained a key trait in plant breeding programs for over half a century (Duvick 2005; Liu et al. 2020; Yang et al. 2021). A deeper understanding of its ecological function, particularly its dynamics under different crop rotation and tillage practices, would significantly advance scientific knowledge. Such knowledge would also inform predictive, model‐based research focused on soybean phenological development (Severini et al. 2024), yield gap estimations, and yield responses to climate stress factors (Setiyono et al. 2007; Bao et al. 2015; Yan et al. 2018; Kothari et al. 2024; Couëdel et al. 2025). Additionally, this insight could also contribute to the broader goals of increasing food production, not by expanding farmland, but by optimizing plant phenotypes to enhance crop ecophysiological processes (Fischer et al. 2014). As precipitation patterns are changing towards wetter springs and drier summers (Grady et al. 2021), it remains unknown how these shifts have interacted with tillage intensity and how this has affected soybean height and productivity over decades of farming.

Herein, we used eight long‐term (50 years) crop production systems to help close the knowledge gap regarding the impacts of crop rotations combined with tillage practices on soybean yields and plant phenes. Additionally, we aimed to explore how weather patterns influence early soybean growth performance, and how consequential early‐season plant height differences are related to yield outcomes. Early detection of possible yield loss via plant height/canopy size offers opportunities to adjust management to mitigate yield losses under stressor conditions (Ordóñez et al. 2015), therefore increasing production. Drones and remote sensing technologies are rapidly advancing in this area. To our knowledge, the most recent study examining rotation and tillage interactions evaluating crop plant height and yield responses focused solely on maize (Boomsma et al. 2010), leaving soybean overlooked. In a 50‐year period, advances in soybean varieties, the use of more modern planting equipment, and climate changes all raise doubts about whether conclusions from earlier time frames are still relevant today.

Our objectives were:

1. Assess the long‐term impact of weather patterns and tillage systems on soybean plant height and yield gains over five decades in monocropping versus soybean in rotation with maize.
2. Identify associations between climatological variables and relative yield and plant height responses to treatment factors.
3. Investigate potential relationships between early‐season plant heights, seasonal weather variables, and yield outcomes under low‐ and high‐yielding conditions.

## 2 Materials and Methods

### 2.1 Experimental Plots Descriptions and Layout

A long‐term (50‐year) tillage and rotation experiment was conducted at the Agronomy Center for Research and Education, Purdue University (ACRE), West Lafayette, Indiana. This ongoing experiment was originally established in 1975 by Purdue University to address soil conservation and economic sustainability concerns for both maize and soybean production. Changing weather conditions during the long‐term trial period enabled the exploration of potentially new impact factors on the relative tillage responses of soybean beyond rotation alone. This experiment encompasses four tillage systems in combination with the most predominant rotation system used within the US Corn Belt (soybean following maize) versus continuous soybean. The historical tillage managements consisted of Fall Moldboard plowing (depth of ~23 cm) + spring secondary tillage, Fall Chisel (depth of 20 cm) + spring secondary tillage, Ridge/Strip‐Till, and No‐Till. Ridge‐Till plots from 1975 to 2009 relied on an annual post‐emergence reshaping (using interrow cultivation with a ridging tool) of the ridges on which the 76‐cm row width soybeans grew. After 2009, ridge plots were transformed into a Fall Strip‐Till system using the identical 76‐cm row width; no spring tillage occurred before planting in either Ridge or Strip‐Till. For simplicity, the entire Ridge/Strip‐Till system will hereafter be referred to as Strip‐Till. Soybean planting date was the same for all tillage systems within a year. Soil fertility levels were maintained well above critical levels with regular macronutrient applications during the study, and lime was applied when recommended.

The field experiment layout consists of a split‐plot design with 4 replications, where the main plot was assigned to rotations and the subplots to tillage systems (Figure S1). Each individual plot unit was 46 m long and 9.9 m wide. Each individual plot unit accommodated 12 rows spaced at 76‐cm row widths planted with a No‐Till‐capable, row‐crop planter. However, soybean row spacing was 19 cm from 1995 through 2004 in all but the Ridge‐Till system as the researchers adapted to the common narrow‐row production systems facilitated by the availability of improved No‐Till drill planting equipment. In 2005, the soybean planting system reverted to the 76 cm spacing as the adoption of narrow‐row soybean production declined in US Midwestern States and as most soybean farmers employed row‐crop planting equipment instead of narrow‐row drill seeding. Before and after this 10‐year timeframe, row spacing was constant for all growing conditions (tillage × rotation combinations).

Many soybean varieties were employed since 1975; the central selection goals were to utilize adapted‐maturity cultivars that were yield‐competitive, above‐average in tolerance to soil‐borne diseases, and high‐performing even in wide‐row production systems. These varieties encompassed different commercial brands and breeding companies. Genetically engineered (GE) soybeans were first used to facilitate post‐emergent weed control in 2000. Glyphosate‐tolerant varieties have been used consistently since then, and more recent varieties (i.e., 2023–2024) also have a dicamba tolerance trait (Table S1). Regardless of the variety traits, all soybean plots were maintained in a near weed‐free condition using residual herbicides and hand weeding when required to avoid confounding yield results with alternate weed competition levels.

### 2.2 Weather Data Sources and Calculations

The long‐term weather data (50 years) was obtained from the National Oceanic and Atmospheric Administration (NOAA 2023) located within 2 km distance from the plots. This data was curated and processed by the Indiana State Climate Office at Purdue University. Our data analysis focused on detecting weather anomalies during spring (1 April to 20 June) and summer (21 June to 20 September) seasons. The designated “spring” period began in April instead of 21 March because soybean plot planting never occurred in March. Weather anomalies were considered as the departure in absolute values from the average of the 50‐year period; these differences were tracked individually for spring and summer seasons from 1975 to 2024. Wet, dry, warmer, and colder years were determined by the relationship between both the calculated average precipitation and temperatures in spring and summer, respectively. This relationship defined quadrants that best characterized mean weather conditions for each individual year.

**FIGURE 1:**
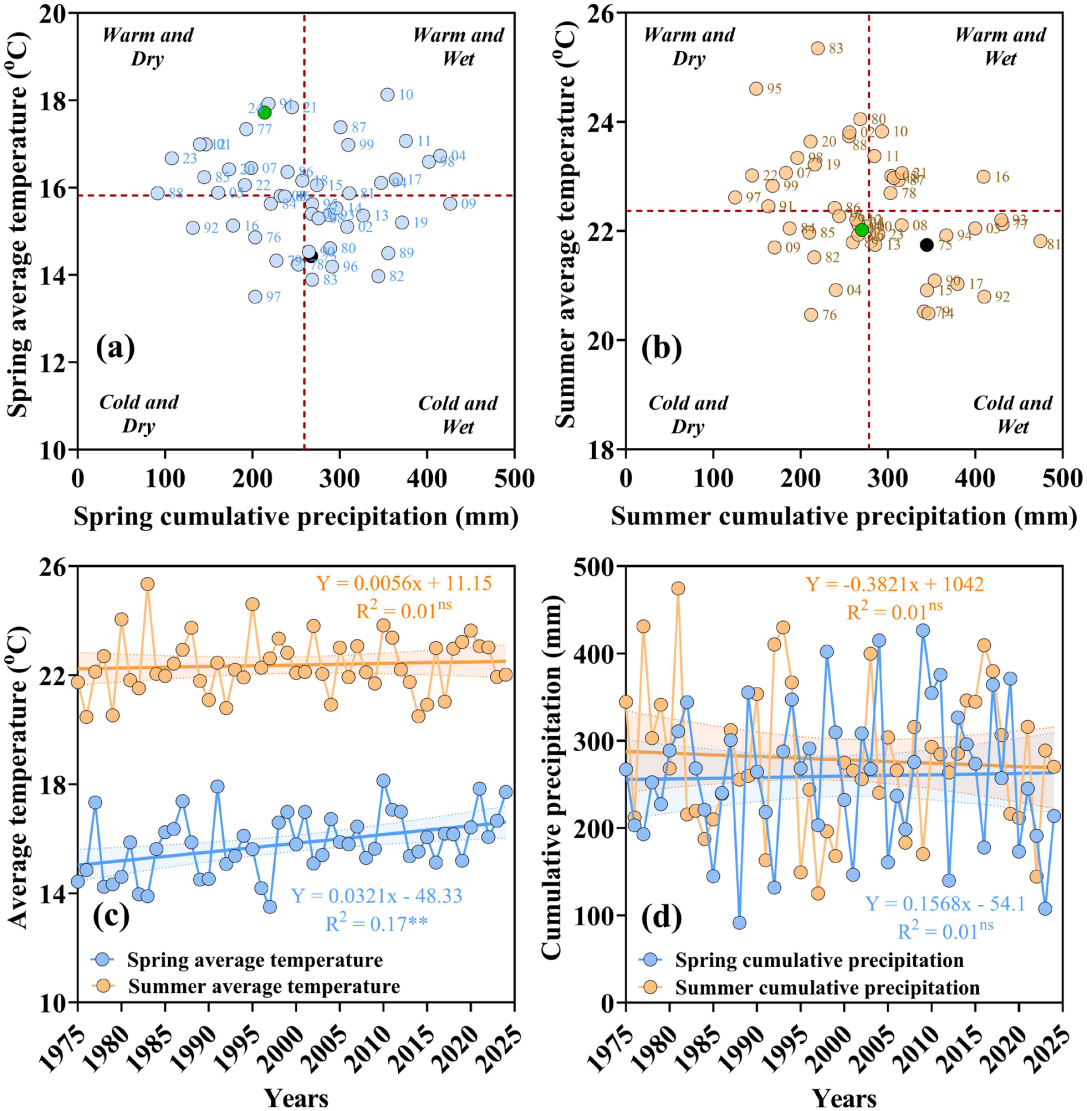
Annual average of spring and summer weather conditions from 1975 to 2024. Panel (a) represents the relationship between annual average cumulative precipitation versus average temperature during spring (1 April to 20 June). Panel (b) represents the relationship between average cumulative precipitation versus average temperature during summer season (21 June to 20 September). Vertical dotted red lines in Panels (a and b) represent the average precipitation or average temperature across 50 years. Points represent each individual experimental year; a black color highlights the first experimental year (1975), and green color the latest experimental year (2024). Panels (c and d) represents the trend averages of temperature and cumulative precipitation for each individual year. Coefficient of determination (*R* ^2^), and the dotted bands indicate the 95% of the confidence interval. Light blue points represent the spring, and orange points the summer season.

Temperature and precipitation trends over time (50 years) were determined by assessing the relationship between the weather variable and the year. Data resolution enhancement was employed for variables such as precipitation (mm), maximum daily air temperature, average daily air temperature (°C) and thermal time (°C days) by analyzing the data in intervals from planting to 4 weeks, and from 4 to 8 weeks after planting. Thermal time was calculated following the standard approach:

(1) $$\mathrm{CTT}=\sum \mathrm{Max}\left(\frac{T_{\max}+\;T_{\min}}{2}-T_{\text{base}},0\right)$$

where *CTT* is the cumulative thermal time (°C day), *T*_max_ and *T*_min_ correspond to maximum and minimum daily air temperatures (°C), respectively, and *T*_base_ is the temperature below which crop growth is assumed to cease (10°C for soybean growth). The Max (…, 0) function is used to constrain negative TT values to zero, thereby accounting for the absence of growth progress below the base temperature. Spring temperature increase was calculated using the parameters of the linear regression equation shown in Figure 1c. This equation is represented as (*y = ax + b*), where *y* = dependent variable, *a* = slope, *x* = independent variable, and *b* = intercept. The increase in temperature (*y*) was calculated when *x =* 1975 and 2024. The spring air temperature gain over the 50‐year period was calculated as the sum of the annual slopes derived from the equation shown in Figure 1c, and is reported in °C.

### 2.3 Planting Date, Plant Height and Yield Data Collection

Planting occurred when weather conditions allowed, and when soil moisture and temperatures were optimal for seed placement and germination (Table S1). Planting date changes over time (50‐year) were assessed by correlating the recorded planting date expressed as day of year (DOY) versus each year. Plant stand count was evaluated following seedling establishment at approximately 4 weeks after planting. Plant population was determined by counting the number of plants within 1 m length of row at five random spots per plot. Pest management practices followed recommended practices for Indiana soybean production.

Soybean plant heights were determined on two different calendar days (approximately at 4 and 8 weeks after planting) each year, and sometimes a few days before or after the scheduled day due to weather challenges. The plant height protocol consisted of randomly selecting 20 representative plants from center rows within individual plots. Plant height was considered the distance from the soil surface to the uppermost expanded trifoliate. Plant height gain was calculated as the actual plant height (cm) divided by the number of days since planting. Height gain was expressed in cm per day^−1^ from planting to 4 weeks or from 4 to 8 weeks after planting.

Yield data was recorded from each tillage and rotation combination. Yield data was taken using plot combines spanning 1.65 m in width for the entire plot length, either harvesting in a single or double pass per plot. Final yield was always reported in kg ha^−1^ at a moisture content of 130 g per kg of grain. It is important to note that yield data from the year 1996 were excluded from our analysis due to crop failure, despite efforts to replant soybeans late in the season to maintain rotation integrity. Yield gain trends over time were evaluated for both soybean–maize rotation and monocropping by averaging data across the four tillage systems. Furthermore, annual average soybean yields for Indiana from the USDA‐NASS (2024) reports were used to compare statewide trends with our long‐term tillage dataset. Additionally, annual yield advantages for each tillage system were quantified as a ratio between yields under rotation relative to the monocropping system. These yield advantages were subsequently averaged over 49 years of the experimental dataset and reported as a %.

Additionally, the yield performance under low‐ and high‐yielding conditions was investigated to discern patterns and variations in yield across individual years. Yield categories were delineated by dividing datasets (tillage × rotation combinations) into quartiles. The first and fourth quartiles for each tillage and rotation combination were selected by sorting the entire 49‐year dataset (i.e., excluding 1996) into years from the lowest to the highest yields, respectively. Each quartile encompassed 25% of the dataset, and quartiles with the lowest and highest yields, respectively, were extracted for detailed analyses of factors influencing variations in yield and plant height. Weather performance was also characterized for each yielding condition.

### 2.4 Data Analysis

Means of each studied variable were calculated across four replications per year. Simple linear regression models were used to assess the relationships between (i) annual precipitation and yield, (ii) yield and plant height, and (iii) plant height and spring precipitation. The goodness of fit for each model was evaluated using the coefficient of determination (*R* ^2^) including their levels of significance (*p* = 0.01, 0.05 and 0.001).

General Linear Mixed models were implemented to evaluate the effects of rotation, tillage, and their interaction on soybean traits. We focus on reporting results related to the factors of interest (rotation, tillage, and two‐way interaction rotation × tillage). Our general mixed models included the following fixed categorical independent factors: rotation (2 levels: soybean after maize and soybean in monocropping); Tillage (4 levels: Moldboard Plow, Chisel, Strip‐Till and No‐Till), and the two‐way interaction (rotation × tillage). Year and replication were treated as random factors. Year was included in the model to control it statistically as a potential source of variation of the independent factor of interest between years. We excluded any interactions with year in the models because trends would be poorly estimated due to frequent changes in soybean varieties, rendering year‐to‐year comparison invalid. Statistical analyses were performed through the PROC GLIMMIX procedure on SAS 9.4 software (SAS Institute). Tukey's studentized range test (HSD) was implemented for pairwise comparisons of means using the LSMEANS statement in PROC GLIMMIX, and statistically significant differences were set at *p* < 0.05. Finally, data visualization and additional analysis were performed using GraphPad Prism version 10.0 for Windows (GraphPad Software, Boston, MA, USA).

## 3 Results

### 3.1 Spring and Summer Weather Trends

Over the 50‐year period of study, we observed significant fluctuations in spring and summer weather patterns, generating various microclimates such as warm‐dry, warm‐wet, cold‐dry, and cold‐wet that soybeans were exposed to early in the growing season (Figure 1a,b). Regression analysis between annual average temperatures and years indicated a consistent warming trend since the middle 1970s, with spring‐season temperatures increasing at the rate of 0.032°C/year, resulting in a total rise of 1.6°C from 1975 to 2024. Average summer temperatures also depicted noticeable anomalies over time, yet the relationship between year and average summer temperature remained relatively stable over time, with no significant changes detected (Figure 1c). Meanwhile, spring‐season precipitation increased annually by 0.16 mm, whereas average summer precipitation declined by 0.38 mm per year (Figure 1d). The warming spring weather conditions facilitated generally earlier soybean planting in later decades (Figure S2).

### 3.2 Plant Density, Plant Height and Yield Across Rotation, Tillage and Its Interactions

The year factor significantly influenced plant phenes and other variables considered in this long‐term experiment (Table S2) as might be expected given the year‐to‐year variability in weather conditions (Figure 1) and changing genetic inputs. The rotation factor significantly affected both plant density and plant height at 4 weeks, with continuous soybean showing slightly higher values compared to rotated soybean (Tables 1 and S2). However, no rotation differences were observed in plant height at 8 weeks or in the accumulated plant height gain between observation periods (Table S2).

**TABLE 1:** Agronomic, morphophysiological phenes and seed yield measured at each tillage and rotation combination across 49 years, excluding 1996.

Source of variation			Plant density (plants m^−2^)	Plant height at 4 weeks (cm)	Plant height at 8 weeks (cm)	Height gain from 4 to 8 weeks (cm)	Seed yield (kg ha^−1^)
Rotation (R)	Soybean‐maize		34.7B	17.2B	54.1A	37.8A	3750A
	Continuous soybean		35.0A	17.6A	54.1A	37.5A	3495B
Tillage (T)	Moldboard Plow		36.1a	18.1a	56.3a	39.1a	3719a
	Chisel		35.4b	17.4b	54.7b	38.2b	3609b
	Strip‐Till		32.4c	17.7b	53.9b	37.6b	3580b
	No‐Till		35.4b	16.3c	51.3c	35.8c	3584b
R × T	Soybean‐maize	Moldboard Plow	36.3a	18.2a	57.3a	40.1a	3854a
		Chisel	35.1bc	17.1 cd	54.4b	38.3b	3712b
		Strip‐Till	32.5d	17.6bc	54.3bc	38.1b	3744b
		No‐Till	34.7c	15.8e	50.3e	35.2d	3692b
	Continuous soybean	Moldboard Plow	36.0ab	18.1ab	55.4b	38.2b	3584c
		Chisel	35.9ab	17.7ab	55.1bc	38.3b	3506d
		Strip‐Till	32.5d	17.9ab	53.7c	37.1c	3416e
		No‐Till	36.1ab	16.8d	52.4d	36.4c	3476de

*Note:* Planting density was assessed at 4 weeks after planting and reported in plants per square meter. Plant height data was collected at 4 and 8 weeks after planting and reported in cm. Yield averages for each tillage and rotation systems are reported in kg ha^−1^ at 13% moisture. Tukey's HSD test was implemented for pairwise comparisons of means with α set at 0.05. Different letters denotates significant differences between means.

Tillage systems significantly impacted plant density and height (Table S2), with Moldboard Plow achieving the highest values in both variables compared to the other tillage systems (Table 1). No‐Till systems had shorter plant height at both measurement times and less height gain between these times. Plant density in the No‐Till system was not significantly different from that in the Chisel but was significantly higher than in Strip‐Till (Table 1). From 1995 to 2004, Ridge‐Till consistently exhibited lower plant density than other tillage systems (Table S3), an outcome that was expected, as Ridge‐Till was the only system planted in wide 76‐cm rows when narrow‐row drilling was employed in the other three tillage systems. The seeding rate target for each tillage system was adjusted for row width and then‐current recommendations but was always consistent within a common row width at planting. Although average plant heights at 4 and 8 weeks were highest in Ridge‐Till, height differences among tilled systems in that 10‐year period were small.

Rotation systems significantly influenced soybean yields (Table S2), with higher values observed in the soybean‐maize production system (3750 kg ha^−1^) compared to continuous soybean (3495 kg ha^−1^). Tillage practices significantly affected soybean yields (Table S2), with Moldboard Plow consistently outperforming other tillage methods. The Moldboard advantage persisted across both continuous (up to 4.4%) and soybean‐maize rotation (up to 4.9%) when compared to other tillage methods (Table 1). The interaction between rotation versus tillage showed that No‐Till yields were comparable to those of other tillage systems (Table 1), although they were 3.1% and 4.4% lower than Moldboard Plow in continuous and rotational systems, respectively (Table 1). Despite the shorter plants in the No‐Till system, yields did not significantly differ from those observed in Chisel and Strip‐Till systems, which are considered intermediate conservation tillage methods.

Interactions between rotation × tillage significantly influenced yield and all analyzed plant phenes (Table S2). Moldboard Plow consistently showed higher absolute phenes values compared to the other combinations in the soybean–maize rotation (Table 1). Note that the No‐Till system exhibited comparable plant density relative to the other combinations but with statistically shorter plant height (Table 1). With continuous soybean, the No‐Till system exhibited shorter plant heights (7.7%) and lower yields (3.1%) than Moldboard Plow, although observed yields with No‐Till did not significantly differ from Chisel and Strip‐Till (Table 1).

### 3.3 Impacts of Rotation and Tillage System Combinations on Yield and Yield Gain Trends Over Time

**FIGURE 2:**
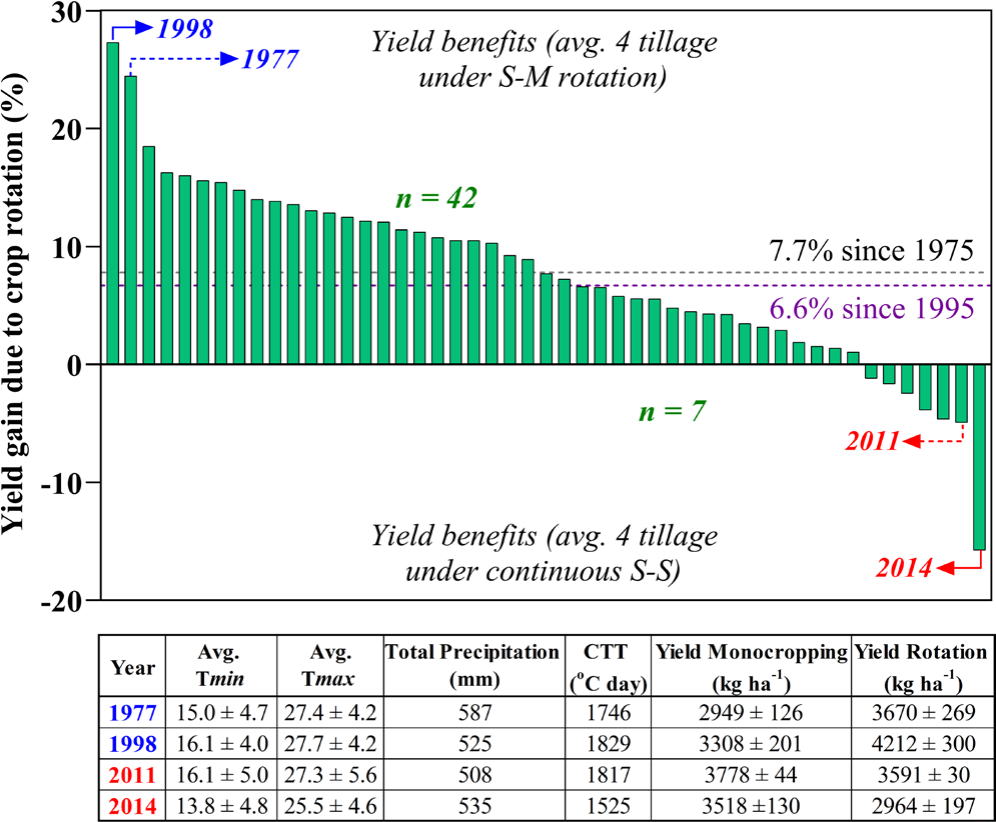
Yield gains due to rotation (or continuous soybean yield penalty) for each individual year. Each bar represents the difference between measured yield (averaged across the four tillage systems) under soybean‐maize rotation minus the yield in continuous soybean expressed as a percentage. Blue color numbers indicate the year with highest proportional yield gains due to rotation, and the red the lowest. The dotted horizontal line indicates the average yield gain, the black color represents the average across 50 years, and the purple the average advantage since 1995. The table shows main weather variables and their performance over the growing season (May 1 through September 30), and average yields from both rotational systems. Note that the 1996 yield result was excluded due to exceptionally late planting.

The overall average yield gain due to rotation from 1975 to 2024 was 7.7% (Figure 2), a level slightly greater than that of 6.6% measured post‐1994. However, these rotation advantages are less than that reported before 1995, when soybean yield advantages due to rotation were first reported for this experiment (West et al. 1996). Although the magnitude of yield benefit varies across years, the yield gain was consistently positive in most studied years (42 of the 49 years), while negative rotation outcomes were experienced in only 7 years (Figure 2). The most substantial negative yield benefits in rotation soybean occurred in 2014, while the highest positive yield gains with rotation soybean were observed in 1998 and 1977 (Figure 2).

Weather conditions may have impacted the most notable differential yield outcomes for rotation systems. The year 1977 was characterized by greater precipitation than average. The year with the highest rotation yield advantage (1998) was characterized by slightly higher minimum and maximum temperatures (and consequently higher CTT) with normal precipitation. The large 16% yield decline for rotation soybean in 2014 occurred in a growing season characterized by low minimum and maximum temperatures and much lower CTT (Figure 2).

**FIGURE 3:**
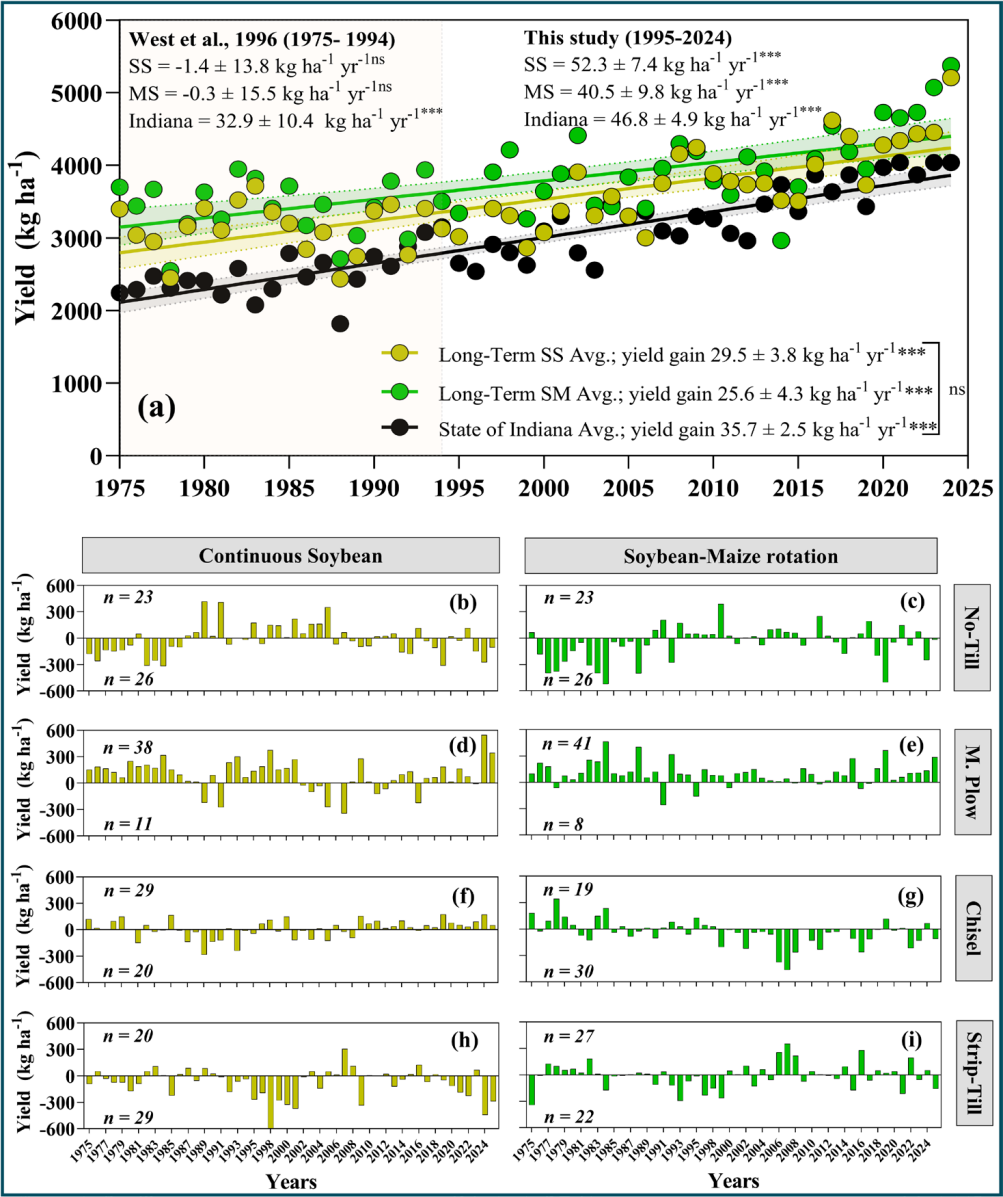
Soybean yield gains over time. Panel (a) represents yield gains over time for Indiana (black points) versus experimental yields (averaged across four tillage systems) in monocropping (gold points) and rotation systems (green points). The dotted lines and shade bands indicate the 95% confidence interval. Panel (b, d, f, h) represents yield gains for each tillage system compared to the overall yield average across four tillage systems in monocropping. Panel (c, e, g, i) represents yield gains for each tillage system relative to the yield average of the four tillage systems in maize‐soybean rotation. Each bar represents the differences between measured yield for the tillage system in each individual year minus the average yield across 4 tillage systems within the rotation system in the same year. The number of years when yields for a tillage treatment were above or below average is indicated in each panel. Each slope is accompained by its corresponding *p*‐value, reported as follows: *** = (*p* = 0.001) and ns = not significant.

Rotation yield advantages varied with tillage. Yield advantage due to rotation was significantly higher for Strip‐Till (10.5%) than for either Chisel or No‐Till (6.2%) and was intermediate for Moldboard Plow (Figure S3). Overall soybean yields increased over time despite no yield gain, or a tendency to decline, during the first 20 years when little change occurred in variety selection (Figure 3a). Soybean monocropping showed a slightly greater rate gain of 29.5 kg ha^−1^ year^−1^ than the 25.6 kg ha^−1^ year^−1^ achieved in rotation systems, but both are lower than the Indiana state average gain of 35.7 kg ha^−1^ year^−1^ (Figure 3a). Individual yield gains for each tillage method within monocropping and rotation systems were slightly different. Monocropping yield gains over time ranged from 28.1 to 30.3 kg ha^−1^ year^−1^, while yield gains in rotation with maize ranged from 21.3 to 29.6 kg ha^−1^ year^−1^. In both cases, annual yield gains were hightly significant (*p* = 0.001). No‐Till monocropping showed the highest gain, while Chisel under rotation systems showed the lowest gain (Figure S3). We found inconsistent yield gain benefits across the 50 years of experimentation when comparing the annual yield for each tillage system versus the overall average of the four tillage systems per rotation (Figure 3, panels b through to i). Numerically, Strip‐Till in continuous soybean and Chisel under maize‐soybean rotation yielded lower than No‐Till in 29 and 30 years out of 49, respectively (Figure 3g,h). Compared to the average of the four tillage treatments, Moldboard Plow generally resulted in a greater number of years of above‐average yield than No‐Till in both rotated (in 41 out of 49 years) and monocropping (in 38 out of 49 years) settings (Figure 3d,e).

### 3.4 Soybean Yield Responses to the Increase in Average Temperature Over the Past 50 Years

**FIGURE 4:**
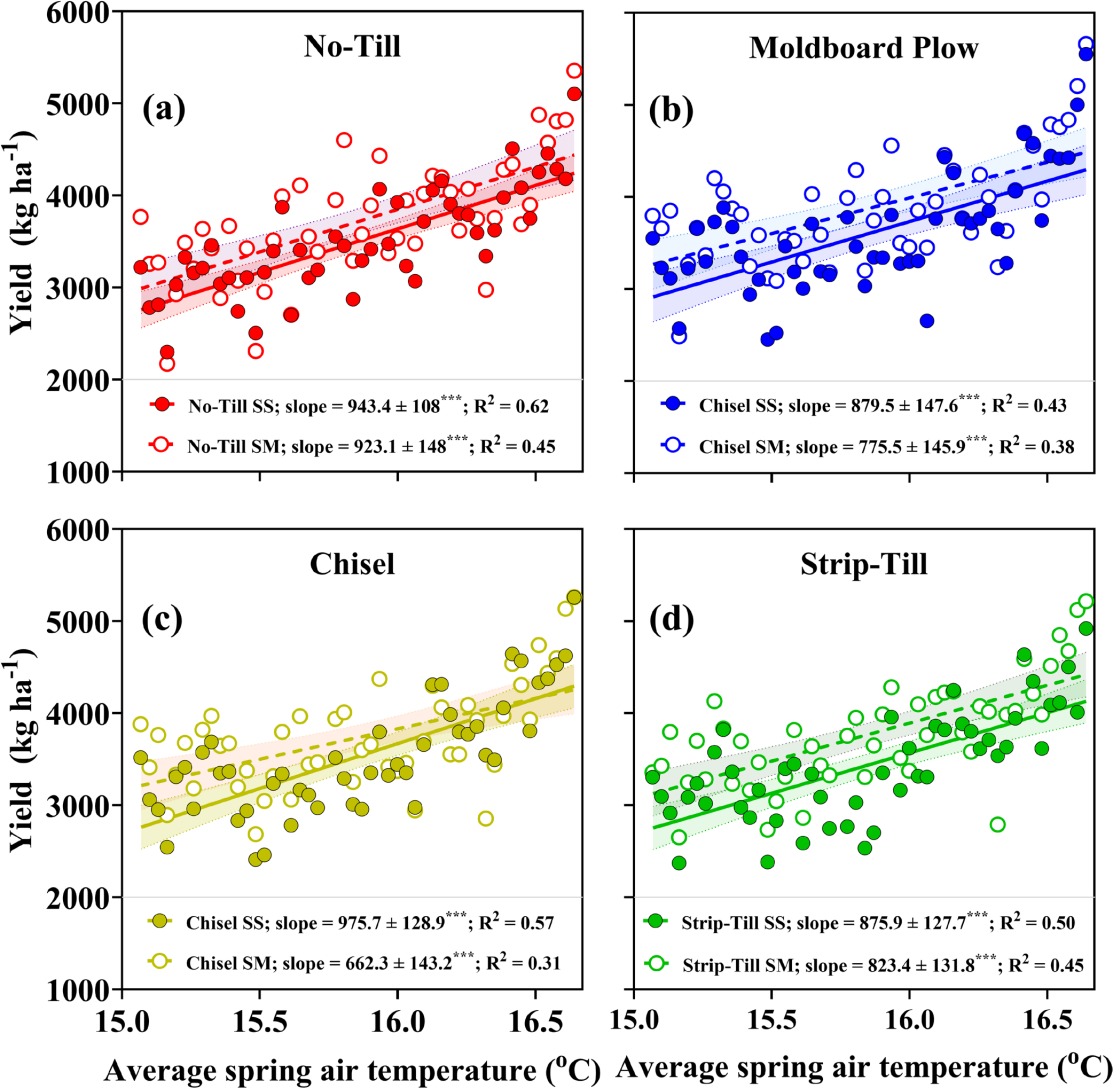
Relationship between the average air temperature during the spring period across 50 years and soybean yields. Filled circles accompanied with solid lines represent soybean monocropping, while open circles with dashed lines indicate soybean‐maize rotation. Red circles correspond to No‐Till system (a), blue to Moldboard Plow (b), yellow to Chisel (c) and green to Strip‐Till (d). The dotted lines and shade bands indicate the 95% confidence interval. Slopes and coefficients of determination (*R* ^2^) for each linear model are provided. Each slope is accompanied by its corresponding *p*‐value, reported as follows: *** = (*p* = 0.001).

Our long‐term analysis across eight soybean production systems (rotation × tillage) showed that yields improved as average air temperatures increased, with highly significant relationships (*p* = 0.001) observed for both rotation systems (Figure 4). The coefficient of determination (*R* ^2^) values ranged from 0.50 to 0.62 for the monocropping system and from 0.31 to 0.45 for soybean‐maize rotation. Across both rotations, yield increases were consistently higher in soybean monocropping compared to soybean‐maize rotation, with slope values indicating that the monocropping system benefited more from rising spring average temperature. Specifically, slope values for soybean monocropping ranged from 876 to 976 kg per unit of temperature increase (Figure 4a–d), whereas soybean‐maize rotation ranged from 662 to 923 (Figure 4a–d). Despite significant yield increases with spring temperature increase across all systems, No‐Till management showed higher slopes and average correlations across rotations (Figure 4a), reflecting potentially enhanced resilience to increasing average spring temperatures.

### 3.5 Relationships of Plant Height at 4 and 8 Weeks With Weather Factors

The relationships between plant height at 4 or 8 weeks after planting versus cumulative precipitation were consistently positive (albeit with relatively low slopes) but rarely significant within tillage and rotation combinations (Figure S4a,b). Significant correlations at 4 weeks were observed only for No‐Till in soybean–maize rotation and for Strip‐Till in both rotation systems (*p* = 0.05). At 8 weeks after planting, a significant correlation was found only for No‐Till in maize–soybean (*p* = 0.05), while all other tillage system relationships remained not significant.

In contrast, the relationships between plant height at 4 weeks and CTT were consistently positive (with steep slopes) and highly significant (*p* < 0.001) in almost every rotation and tillage combination (Figure S4c). At 8 weeks, Moldboard Plow was the only tillage system that showed no significant height correlation with CTT in either rotation system (Figure S4d), while the rest were all significant.

**FIGURE 5:**
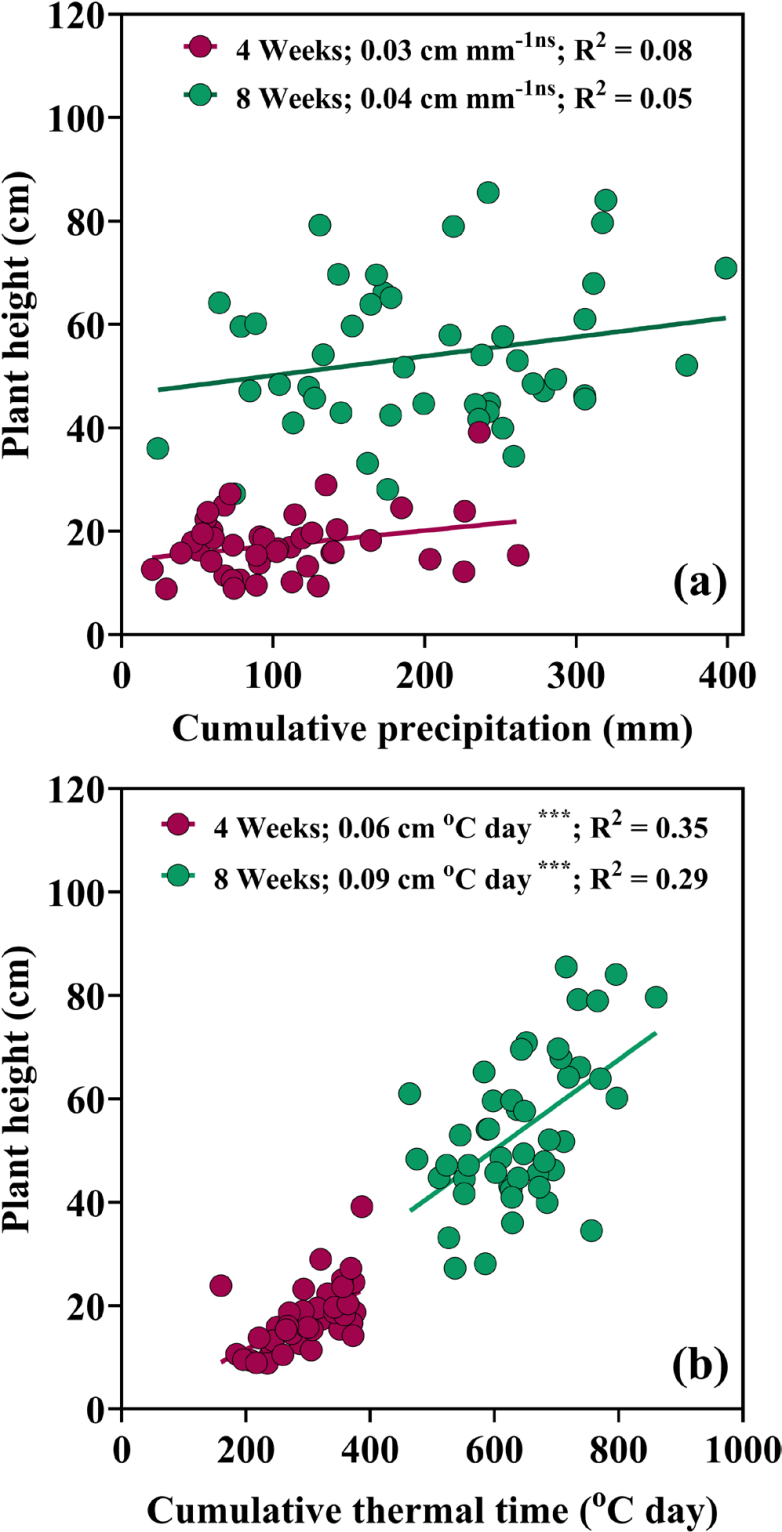
Relationship between soybean plant height, precipitation, and thermal time. Panels (a) and (b) shows the relationship trend line between annual plant height average in (cm) and accumulated precipitation (mm) at 4 and 8 weeks after planting. Panel (b) displays the relationship between plant height and accumulated thermal time (°C day) at 4 and 8 weeks after planting. The slope and coefficient of determination (*R* ^2^) from linear regression analysis are reported with their respective significance levels. Each slope is accompained by its corresponding *p*‐value, reported as follows: *** = (*p* = 0.001) and ns = not significant.

In summary, when averaging across rotation and tillage combinations, thermal time had a stronger influence on soybean height than precipitation, and this effect was consistent at both measurement times (4 and 8 weeks). Although taller plants were observed with increased precipitation, this relationship was not statistically significant (Figure 5a). Soybean height was highly responsive to thermal time accumulation as anticipated, with a strong and highly significant (*p* = 0.001) relationship (Figure 5b). Ranges in accumulated spring precipitation did not significantly improve soybean heights. Specifically, soybean monocropping showed a positive slope of 0.63 kg mm^−1^ of received rain, while soybean under rotation showed a negative slope of −0.4 kg mm^−1^ (data not shown). Most notably, although soybean height varied widely within measurement times (Figure 5), this variability did not result in remarkable differences in final yield, either in relation to rotation or tillage intensity, over the 50 years of experimentation.

### 3.6 Explanatory Variables for Yield and Height Gain Rates in Low‐ and High‐Yielding Conditions

Weather characterization of both low‐ and high‐yielding conditions showed nearly similar performance at 4 weeks. However, from 4 to 8 weeks, the high‐yielding years had 9.3% more precipitation, 6.2% more CTT, plus a 7.5% and 3.6% increase in minimum and mean air temperatures, respectively (Table S4). Averaging data across years, the period from 4 to 8 weeks showed a 4% increase in precipitation, 27% more CTT, and a 15.8% increase in mean temperatures than the period from sowing to 4 weeks (Table S4). Soybean yields from rotation and tillage combinations in both the highest and lowest quartiles from the 50‐year period were not strongly impacted by planting date or CTT accumulated by the end of September (Figure S5a,b). Although both positive and negative effects were observed, none of the relationships were statistically significant (Figure S5a,b). These yield groups for most tillage treatments were also little impacted by growing season precipitation. However, No‐Till soybean responses in the high‐yield quartile for the maize‐soybean rotation were negatively affected by cumulative precipitation (Figure S5a,b).

Plant height gains from planting to 4 weeks showed a significant positive correlation with current planting date and CTT in most cases (Figure S5a,b), and that was consistent in continuous soybean and under rotation. It was interesting that the relationship was more noticeable under low‐yielding conditions. Additionally, height gain per day^−1^ from 4 to 8 weeks was also positively and significantly correlated with planting date and CTT (Figure S5a,b) in both systems. As with the early growth period, cumulative precipitation did not correlate with plant height gain day^−1^ during this time frame regardless of yielding condition (Figure S5a,b).

## 4 Discussion

### 4.1 Long‐Term Rotation and Tillage Effects

Strategic soybean rotation with maize and adaptative tillage practices, particularly less intensive tillage, can significantly boost soybean yields and resilience. This study provides novel findings into the role of rotation and tillage management practices for soybean production systems amidst rising temperatures and shifting precipitation patterns by delineating 50‐year trends in yields and mean seasonal temperatures, while quantitatively linking productivity (Figures 3 and 4). The potential of a monocropping system (i.e., > 10 years, and in our case 50 years) to inform future soybean production was previously unknown, as such comprehensive data are not available in the literature. These decadal trends for rotation and tillage practices under changing weather conditions can inform breeding programs focused on improving crop resilience and train models to better predict future growing scenarios in the context of climate change and sustainability goals.

Resilience in agriculture is not solely defined by maximum output, but by the ability to adapt and thrive under shifting conditions. No‐Till system performance in this study confirmed that sustainability and productivity can coexist even when traditional methods like Moldboard Plow yield more in the short term (Figure S3). Our No‐Till yields were comparable with the intermediate tillage systems, suggesting its potential for greater resilience. Although Moldboard plow consistently produced higher yields, these gains likely reflect legacy soil conditions rather than a sustainable yield advantage under modern climate and management scenarios. The 7.7% average yield advantage we observed for soybean rotated with maize (Figure 2) was lower than the 8% to 25% range reported from medium to long‐term studies conducted from 6 to 28 years (Crookston et al. 1991; Lund et al. 1993; Pedersen and Lauer 2003). Seifert et al. (2017), in their analysis of 319,815 rainfed and 16,751 irrigated geolocated soybean fields within the US Corn Belt, reported yield penalties of 10.3% and 9.3%, respectively, for soybean after soybean compared to rotation systems, though these findings were based on only 6 growing seasons where there was no information available on the attendant tillage systems and on whether monocropping soybean was employed for a 2‐year or longer period.

Another regional projection of soybean yields by 2070 suggests that warming climates are expected to enhance the benefit of crop rotation by 8.4% to 13.8% across the Corn Belt (Zhou et al. 2024). However, it is noteworthy that when soybean is rotated with other crops in less productive environments the yield benefit can be more pronounced, as was shown by Simão et al. (2023) who found a 35% rotation advantage in a 44‐year experiment with continuous soybean versus soybean after winter wheat and sorghum in Kansas' silty loam drylands. This yield benefit is insightful for less productive zones, because in their study, No‐Till soybean rotated with winter wheat or sorghum achieved the highest yield of 2800 kg ha^−1^ in a region with more frequent precipitation deficits. Interestingly, our No‐Till yields both under rotation and monocropping systems far surpassed the best average yields observed in Kansas. Furthermore, understanding the role of tillage operations is critical for guiding investment decisions, as total tillage practices account for $3.9 billion in annual expenditures in US agriculture (USDA‐NASS 2024).

In the US Midwest, soybean rotated with maize is the dominant cropping system. Continuous soybeans are mostly utilized in South America. However, the current cropping system landscape may change in response to policies. For example, policies released by the US Congress (2022), such as the Inflation Reduction Act section 45Z in the United States (Public Law 117–169, 136 Stat. 1818) provide tax credits for less carbon intensive practices given the cost of increased biodiesel production to meet environmental goals. This could potentially increase demand for more soybean production and hence increase soybean area, potentially including continuous soybean systems. By providing 50‐year continuous soybean yield penalties by individual tillage systems, our results could reliably inform decision makers and subsequent economic analysis of soybean production systems in the USA.

### 4.2 The Impacts of Tillage Methods on Plant Height and Soil Pools

Optimal agricultural practices may not always align with traditional expectations of higher yields, as demonstrated by the observation that taller plants in plowed soils did not lead to significant higher yields (Table 1). A more nuanced approach is needed to rethink and redesign global soybean production systems for long‐term sustainability and resilience. While initial plant heights differed between systems, soybean growth displayed such inherent growth flexibility that only small yield differences occurred between treatments with initially shorter versus taller plants (Table 1). This implies that other factors, such as long‐term soil health, nutrient availability, and weather conditions during the growing season, play a more critical role in determining the final yield outcomes. While conventional tillage can provide more optimal conditions for early soybean growth (Sangotayo et al. 2022), soybean plant growth in No‐Till systems may face challenges, including cooler soil temperatures that delay seed emergence and excessively moist soils or dry conditions in spring that can negatively impact root function (Dwyer et al. 1996). These factors can contribute to shorter initial plant height (Vyn et al. 1998; Ball‐Coelho et al. 1998), but these can be overcome when later‐season growing conditions are more favorable.

Although comprehensive root studies in soybean under long‐term experiments remain underdeveloped (Ordóñez et al. 2020; Nichols et al. 2024), our preliminary root depth data measured at 4 weeks post‐planting suggest that the root system's ability to explore deeper soil profiles is influenced by rotation and tillage methods (Figure S6), which align with results shown by Nichols et al. (2019). Tall soybean plants may be associated with deeper root system and greater root growth; however, increased height does not guarantee higher yields, possibly due to shifts in the source‐sink relationship or root‐to‐shoot ratios (Ordóñez et al. 2020).

Intensive tillage not only influences soybean plant performance but also can alter soil C and N pools. An earlier study, conducted only in the continuous maize and maize‐soybean rotations after the first 28 years of this experiment (Gál et al. 2007), found that Moldboard Plow negatively impacted soil organic C (SOC) and N levels in the top 15 cm compared to No‐Till, but had little negative consequence on cumulative SOC to a 1.0 m depth when measured on a soil equivalent‐mass basis. The same experiment was tested after 46 years including, for the first time, the continuous soybean treatment (in addition to continuous maize and maize‐soybean) and three tillage systems (Moldboard Plow, Chisel and No‐Till). In the 2022 sampling, once again to a 1.0 m depth, No‐Till showed a clear cumulative SOC mass advantage relative to Moldboard Plow and Chisel (Gautam et al. 2025). However, it was striking that no significant differences in cumulative SOC were observed among rotation systems revealing that the soybean monocropping system was not significantly lower in overall SOC or N retention down to a 75 cm soil profile than the maize‐soybean rotation (Gautam et al. 2025). Deep‐soil enrichment of stored C and N, when accomplished by long‐term No‐Till, enhances resilience to climate variability and supports long‐term productivity. Plant and soil health and ecosystem services can be compromised by aggressive tillage even when yields are maximized (Table S2). A shift toward a less intrusive No‐Till strategy combined with other management practices could maintain soybean productivity while fostering long‐term environmental benefits, thus enabling a more sustainable intensification and narrowing yield gaps in global agriculture (Pittelkow et al. 2015; Bowles et al. 2020).

### 4.3 Soybean Yield Progress Over Time

Annual yield gains have increased sharply since 1995, reaching absolute yield gains of 52.3 kg ha^−1^ year^−1^ for soybean in monocropping and 40.5 kg ha^−1^ year^−1^ for rotation with maize. In contrast, yield gain rates from 1975 to 1994 (Figure 3) showed a slight loss over time in both continuous and rotational systems (West et al. 1996). However, over the entire 50‐year period (Figure 3), our yield gains were close to the 23 kg ha^−1^ year^−1^ increase observed in soybean maturity groups III and IV after 80 years of breeding (Rincker et al. 2014). These gains were also comparable to the U.S. national soybean yield increase of 26 kg ha^−1^ year^−1^ over a century of improvement (Egli 2023). The significant shift compared to West et al. (1996) could reflect the limitations of previous farming practices (Table S1) plus the inherent advantages accruing from superior stress‐tolerant varieties and improved management practices in recent decades. Also, these remarkable yield gain values correspond with the introduction of genetically engineered glyphosate‐tolerant cultivars (Table S1), which played a crucial role in advancing U.S. soybean seed yields (Padgette et al. 1995; Rowntree et al. 2013). Growing concerns about food security and the likely impact of environmental changes on food production have injected a new urgency to accelerate genetic gains in breeding programs (Tester and Langridge 2010). Our long‐term study confirmed that, although the annual soybean yield gains remain modest, employment of modern soybean varieties enhances yield resiliency compared to historical cultivars, even under less productive conditions (Figure 3). This suggests that soybean yields will continue to rise unless there is a drastic shift in weather patterns.

The rate of soybean yield increases in our experiment (25.6 kg ha^−1^ year^−1^) was 30% lower than the state level estimate (Figure 3). This is reasonable because state level estimates account for a large geography and soil improvement over the years (e.g., subsurface drainage) as well as planter equipment enhancements to facilitate earlier state‐level planting dates (Deines et al. 2023). Future studies could use this dataset and delineate the contribution of each factor similar to Rizzo et al. (2022). Another interesting observation from this long‐term experiment is that the continuous soybean yield penalty is closing over the years. This is evident by the 14% higher rate of yield increase in continuous soybean than in soybean after maize. This is probably attributed to improved genetics and the use of modern soybean varieties with enhanced disease tolerance and widespread use of seed treatments which may lead to mitigated disease pressure (Rowntree et al. 2013; Lin et al. 2022). This result is of importance in future crop systems designs. Lastly, the 50‐year experimental dataset shows no sign of yield plateau in any of the eight cropping systems which is in itself a positive outcome.

### 4.4 The Increased Spring Temperature in the Last 50 Years Favors Soybean Yields

Local weather data confirmed a 1.6*°*C increase in spring average air temperatures and a trend toward wetter conditions (Figure 1), generally favoring soybean yields (Figures 3 and 4). Our long‐term in vivo results align with previous modeling studies, suggesting that by 2050 soybean, as a C3 plant, will benefit from the increase in air temperatures, ambient CO_2_ levels, and precipitation (Lobell and Gourdji 2012; Bao et al. 2015; Ainsworth and Long 2020). Under these enhanced conditions, rainfed yields would rise by 8% to 35%, unless other extreme abiotic factors disrupt the growing season (Jin et al. 2017; Ainsworth and Long 2020). A field study in Illinois under CO_2_ enrichment suggested that soybean yield may increase by 15%, mainly by converting energy into biomass rather than improving light interception efficiency (Dermody et al. 2008). The combination of a 1.6°C increase in spring air temperatures and current CO_2_ levels likely has contributed to the positive yield trends observed in this long‐term study, independently of rotation and tillage system (Figures 3 and 4). Our yield trends are expected to persist as atmospheric CO_2_ levels continue to rise through mid‐century and beyond (Houghton et al. 2001). Therefore, future modeling efforts that rely solely on rotation practices to predict regional and global soybean productivity dynamics (Seifert et al. 2017; Zhou et al. 2024; Hoffman et al. 2020) would need to account for the effect of elevated air temperature linked to both climate change and tillage intensity for more accurate yield projections.

Our results show that for every 1°C increase in spring temperature, soybean yields increase by 796 kg ha^−1^. While this is confounded by changes in varieties and management, the slope of the regression plot (Figure 4) is high and calls for further research to explore potential opportunities for higher soybean yield gains in the future. The slope was 13% higher in continuous soybean than soybean‐maize, which may imply that soybean in monocropping may be better suited to warmer environments. In fact, while soybean monocropping showed a positive association between yield and average spring precipitation, suggesting increased rainfall may partially alleviate production constraints in continuous systems, soybean grown under rotational systems demonstrated a contrasting, negative relationship (0.63 vs. −0.4 kg mm^−1^, respectively). These findings highlight the critical role of temperature and thermal time unit accumulation in driving crop yield in unique production systems, a focus of recent modeling research endeavors (Setiyono et al. 2007; Sun et al. 2022; Severini et al. 2024). The latter reinforces our earlier claim and sheds light on the complex interactions between crop management and climate variables.

### 4.5 The Potential Benefit of Weather Shift and the Correlation With Soybean Yields and Plant Height

Our long‐term shifts in seasonal air temperature and precipitation (Figure 1) are consistent with previous studies by Dai et al. (2016) and Hamed et al. (2021), who reported increased early‐season precipitation but a decrease in the late‐season rainfall across 8 of the 12 states in the U.S. Corn Belt. From a farming standpoint, if the projected warming trends continue, and spring soil moisture remains manageable (Grady et al. 2021), early soybean planting could become more feasible, as observed in this study (Figure S2). Yield gains are associated with early planting; Faé et al. (2020) reported a linear yield decrease of approximately 45 kg ha^−1^ per day of planting delay in Pennsylvania, U.S. In Ohio, when water was not a limiting factor, soybean planted in April and May yielded 8% to 26% more than those planted in June (Colet et al. 2023). Although we did not specifically assess optimal planting dates (Figure S2), our findings support the yield benefits of early planting, as reported in Indiana (Casteel 2023) and the U.S. Midwest (Mourtzinis et al. 2019) that are facilitated by warmer spring temperatures.

Despite weather changes during our study, we found no direct link between annual yields in our rotation/tillage combinations and the cumulative precipitation or CTT in spring and summer time periods. The lack of a relationship between rain and yield is not surprising. This is because much of the eastern US Midwest has shallow water tables that influence soil water supply to the crop and hence yields (Ordóñez et al. 2018; Rizzo et al. 2018; Elli and Archontoulis 2023; Deines et al. 2024). However, novel and significant relationships emerged between yield and the spring temperature increases over the past 50 years. In contrast, a former regional analysis in the U.S. Corn Belt found that overall soybean yields (examined independently of rotation and tillage) were negative and significantly influenced by July maximum temperatures, August minimum temperatures, and July–August total precipitation (Hatfield et al. 2018).

Weather variables such as temperature and precipitation correlated with plant height, indicating that environmental conditions significantly influence early growth patterns (Figure S5). The observed relationship suggests that weather variables can serve as reliable predictors of plant height, offering valuable insights for optimizing crop management strategies. These findings highlight the potential for integrating weather data into agricultural practices to improve yield forecasting and management strategies. As soybean yields are not explained by plant height, future research should investigate the underlying mechanisms driving plant height and weather associations to further enhance predictive models.

While we found no correlation between the final annual soybean yield and 4 to 8 weeks plant height measurements, with a coefficient of determination *R* ^2^ < 0.03 for both systems (data not shown), plant height is an important plant trait, which is routinely measured in breeding programs (Liu et al. 2020; Yang et al. 2021). Plant height correlates with node appearance rate and hence could facilitate phenology prediction, a trait of interest to many disciplines including crop adaptation to future environments (Yang et al. 2021) or canopy cover for light interception or weed suppression.

### 4.6 Tillage Impacts Ecological Balance

No‐Till and Moldboard Plow had similar actual plant densities (Table S2), suggesting that No‐Till may be as effective as conventional tillage in maintaining plant population under soybean‐maize rotations. This finding contrasts with previous research suggesting that monocropping production systems may promote harmful root and foliar microorganisms, potentially reducing yields due to plant mortality in moist soil (Kladivko 2001; Neupame et al. 2021; Hao et al. 2021). Despite the potential for reduced yields due to increased pathogen microbial activity in monocropping soil, an earlier study in the same field experiment indicated that Moldboard Plow systems may enhance pathogen populations, such as *Heterodera glycines*, when soybean follows maize (Westphal et al. 2009). This discrepancy suggests that soil health impacts may differ between tillage systems and that tillage practices do not always lead to predictable outcomes in microbial and nematode dynamics. The use of modern soybean varieties with enhanced disease tolerance and widespread use of seed treatments may lead to mitigated disease pressure (Rowntree et al. 2013; Lin et al. 2022), explaining the lack of significant differences in plant density between tillage methods across rotation systems. Overall, our long‐term soybean analysis across eight production systems supports our conclusion that breeding advancements and pest management can reduce the presumed negative impacts of tillage choice on soybean productivity.

Our comprehensive 50‐year dataset could facilitate future crop model calibration and application studies to provide insight into soil and plant processes behind the observed yield increases (e.g., Setiyono et al. 2007; Salmerón et al. 2017; Kothari et al. 2024; Baum et al. 2023). The high‐quality management records (Table S1) associated with the two rotations and four tillage management systems in a factorial design make it ideal for modeling purposes. If the models are able to replicate temporal trends (Figure 3) and spring temperature to yield relationships (Figure 4), then confidence could grow in using these models in future climate change impact studies (Bao et al. 2015; Dai et al. 2016; Hoffman et al. 2020; Hamed et al. 2021; Elli and Archontoulis 2023). Furthermore, the different rotation systems coupled with different tillage practices could help detect model capacity in simulating residue decomposition, an under‐looked process of high importance for system analyses.

## 5 Conclusions

This study evaluated a 50‐year combined impact of climate, crop rotation, and tillage on soybean yield and plant height in West Lafayette, IN. Our long‐term results showed higher soybean yield gains post‐1994, coinciding with more frequent changes in soybean varieties. Since 1994, the rate of yield gain increased significantly, reaching 40.5 kg ha^−1^ year^−1^ in rotation with maize and 52.3 kg ha^−1^ year^−1^ in monocropping. While monocropping soybean achieved the highest yield gain rates, only No‐Till management sustained those gains with consistency over time under both rotational systems, adding to our understanding of tradeoffs in higher gains versus greater stability in long‐term production systems under changing climate. Our study confirmed the critical role of soybean‐maize rotation, with a 7.7% yield advantage over monocropping. Although annual yield differences among tillage treatments were inconsistent across both rotation and monocropping systems, Moldboard Plow yields were generally higher than the other tillage systems. No‐Till yield reductions were not because of any reduction in plant density or early‐season plant heights relative to tilled systems. Our long‐term climatological analysis showed a shift towards warmer and wetter spring conditions, potentially extending the soybean planting window in this region. Despite weather fluctuations, variables like cumulative precipitation and thermal time (°C day) during spring and summer periods did not explain annual yield variation. However, the 1.6°C increase in spring air temperatures improved soybean productivity across all tillage regimes, with a most noticeable improvement observed in the No‐Till system. Plant heights at 4‐ and 8‐weeks post‐planting were more influenced by spring air temperatures than by precipitation, regardless of soybean yield levels. Thus, there is still considerable opportunity to adopt novel management technologies and superior crop varieties to enhance future adaptation strategies for soybean production to climate change that will mitigate environmental concerns.

## Author Contributions

**Raziel A. Ordóñez:** data curation, formal analysis, methodology, validation, visualization, writing – original draft, writing – review and editing. **Shaun N. Casteel:** methodology, resources, writing – review and editing. **Rachel H. Stevens:** data curation, methodology, resources, writing – review and editing. **Sotirios V. Archontoulis:** conceptualization, writing – review and editing. **Tony J. Vyn:** conceptualization, data curation, funding acquisition, investigation, methodology, project administration, resources, supervision, validation, writing – original draft, writing – review and editing.

## Conflicts of Interest

The authors declare no conflicts of interest.

## Supporting information


**FIGURE S1.** Experiment layout and soil characteristics. Continuous soybean and rotated with maize plots are depicted in this figure.
**FIGURE S2.** Planting date patterns over time. Green points show the day of year (DOY) *Y*‐axis and actual day of planting for each year of experimentation on *X*‐axis. Black line is the adjusted linear plateau model *y* = *β*_0_ + *β*_1_𝑥, if 𝑥 ≤ 𝑥𝑠, where: *y* is DOY, 𝑥 is the year, 𝑥𝑠 is the breakpoint, *β*_0_ is the intercept and *β*_1_ the slope. The line's left portion indicates the experiment's average planting date from 1975 to 2008 (18 May), and the blue dotted line indicates a transition point in planting day trends.
**FIGURE S3.** Soybean yield gain over time and yield advantage due to rotation in each tillage method. Annual soybean yield was regressed with years for continuous soybean (SS) and soybean rotated with maize (SM). Tillage systems are denotated as follow: No‐Till in red, Moldboard Plow in blue, Chisel in gold, and Strip‐Till in green. Annual yield advantages were calculated for each tillage systems as a ratio of rotation relative to monocropping system and reported as a %. Standard error bars were calculated across the 50‐year time serie to represent overall variability. The comparison of means was subjected to Tukeys' HSD test. Different letters indicate significant differences, with corresponding *p*‐values set at 0.05.
**FIGURE S4.** Trend lines of the relationship between plant height, precipitation, and thermal time for the eight soybean production systems. Panels (a) and (b) shows the relationship trend‐line between annual plant height average in (cm) and accumulated precipitation (mm) at 4 and 8 weeks after planting. Panels (c, d) displays the relationship trend‐line between plant height and accumulated thermal time (°C day) at 4 and 8 weeks after planting. The coefficient of determination (*R* ^2^) from linear regression analysis is reported with its respective significant levels. Statistical significant levels are indicated by *p*‐values, reported as follows: * = (*p* = 0.05), ** = (*p* = 0.01), *** = (*p* = 0.001) and ns = not significant.
**FIGURE S5.** The relationships of yield, height gain per day at 4, and from 4 to 8 weeks versus management and weather variables. Low‐ and high‐yielding conditions were determined by the 1st and 4th quartiles after ranking from low to high yields (*n* = 12), respectively. Coefficients of correlation (*r*) for final yield (a and b), plant height gain in cm per day from planting to 4 weeks (a and b), and plant height gains between 4 and 8 weeks after planting (a and b) with planting date, thermal unit accumulation (°C day) and cumulative precipitation (mm) for distinct time periods and yielding conditions for each tillage system under continuous soybean and soybean‐maize rotation systems. Tillage systems are abbreviated as follows: CH, Chisel; MP, Moldboard Plow; NT, No‐Till; and ST, Strip‐Till. Bars with positive values indicates that the slope on this regression was positive; negative bars reflect a negative regression value. Horizontal dotted lines in each panel indicate the levels of significance. When a bar reaches either of the horizontal dotted lines, it indicates that the relationship is significant at this level. Statistical significant levels are indicated by *p*‐values, reported as follows: (*p* = 0.10), (*p* = 0.05) and (*p* = 0.001).
**FIGURE S6.** Maximum soybean root depth in‐row and between two rows. Rooting depth (cm) measured at 4 W after planting in monocropping and soybean after maize systems across four tillage methods in year 2024.
**TABLE S1.** Soybean varieties used, planting and harvest dates. G letters indicates maturity groups.
**TABLE S2.** Statistical outcomes from the general linear mixed models for plant phenes (plant heights in cm), plant density (plants m^−2^) and seed yields (kg ha^−1^).
**TABLE S3.** Agronomic, morphophysiological traits and seed yields measured in each tillage and rotation combination from 1995 to 2004 when soybeans were drill‐seeded in 19‐cm row widths for Moldboard Plow, Chisel and No‐Till systems while Ridge‐Till was seeded in 76 cm row widths, as had been the standard practice for all tillage systems before 1995 and after 2004. Plant density was assessed at 4 weeks after planting and reported in plants per square meter. Plant height data was collected at 4 and 8 weeks after planting and reported in cm. Yields averages for each tillage and rotation systems are reported in kg ha^−1^ at 13% moisture. Tukeys' HSD test was implemented for pairwise comparisons of means with α set at 0.05. Different letters denotates significant differences between means.
**TABLE S4.** Weather variables measured across different time periods. Weather data include the cumulative precipitation (mm) and thermal time (°C day), the mean of both minimum and maximum temperature (°C), alongside the mean of these temperatures. Corresponding standard deviations (*n* = 12) are provided for all weather values.

## Data Availability

The data that support the findings of this study are openly available in the Purdue University Research Repository (PURR) at https://purr.purdue.edu/publications/4928/1. 10.4231/TH1A‐M536.
