# Supplementary material for: Climate, Rotation, and Tillage Impacts on Soybean Yield Gains in a 50‐Year Experiment
Source: Glob Chang Biol. 2025 Sep 8;31(9):e70469. doi: 10.1111/gcb.70469 (PMC12417934; doi:10.1111/gcb.70469)
Supplement: Supplementary file 1 — FIGURE S1. Experiment layout and soil characteristics. Continuous soybean and rotated with maize plots are depicted in this figure. FIGURE S2. Planting date patterns over time. Green points show the day of year (DOY) Y‐axis and actual day of planting for each year of experimentation on X‐axis. Black line is the adjusted linear plateau model y = β 0 + β 1𝑥, if 𝑥 ≤ 𝑥𝑠, where: y is DOY, 𝑥 is the year, 𝑥𝑠 is the breakpoint, β 0 is the intercept and β 1 the slope. The line's left portion indicates the experiment's average planting date from 1975 to 2008 (18 May), and the blue dotted line indicates a transition point in planting day trends. FIGURE S3. Soybean yield gain over time and yield advantage due to rotation in each tillage method. Annual soybean yield was regressed with years for continuous soybean (SS) and soybean rotated with maize (SM). Tillage systems are denotated as follow: No‐Till in red, Moldboard Plow in blue, Chisel in gold, and Strip‐Till in green. Annual yield advantages were calculated for each tillage systems as a ratio of rotation relative to monocropping system and reported as a %. Standard error bars were calculated across the 50‐year time serie to represent overall variability. The comparison of means was subjected to Tukeys' HSD test. Different letters indicate significant differences, with corresponding p‐values set at 0.05. FIGURE S4. Trend lines of the relationship between plant height, precipitation, and thermal time for the eight soybean production systems. Panels (a) and (b) shows the relationship trend‐line between annual plant height average in (cm) and accumulated precipitation (mm) at 4 and 8 weeks after planting. Panels (c, d) displays the relationship trend‐line between plant height and accumulated thermal time (°C day) at 4 and 8 weeks after planting. The coefficient of determination (R 2) from linear regression analysis is reported with its respective significant levels. Statistical significant levels are indicated by p‐values, rep [file GCB-31-e70469-s001.docx]

**Supporting information for:** Manuscript ID: GCB-25-1616.R1

**Title:** Climate, rotation, and tillage impacts on soybean yield gains in a 50-year experiment.

**List of Authors:** Raziel A. Ordóñez, Shaun N. Casteel, Rachel H. Stevens, Sotirios V. Archontoulis, Tony J. Vyn

**
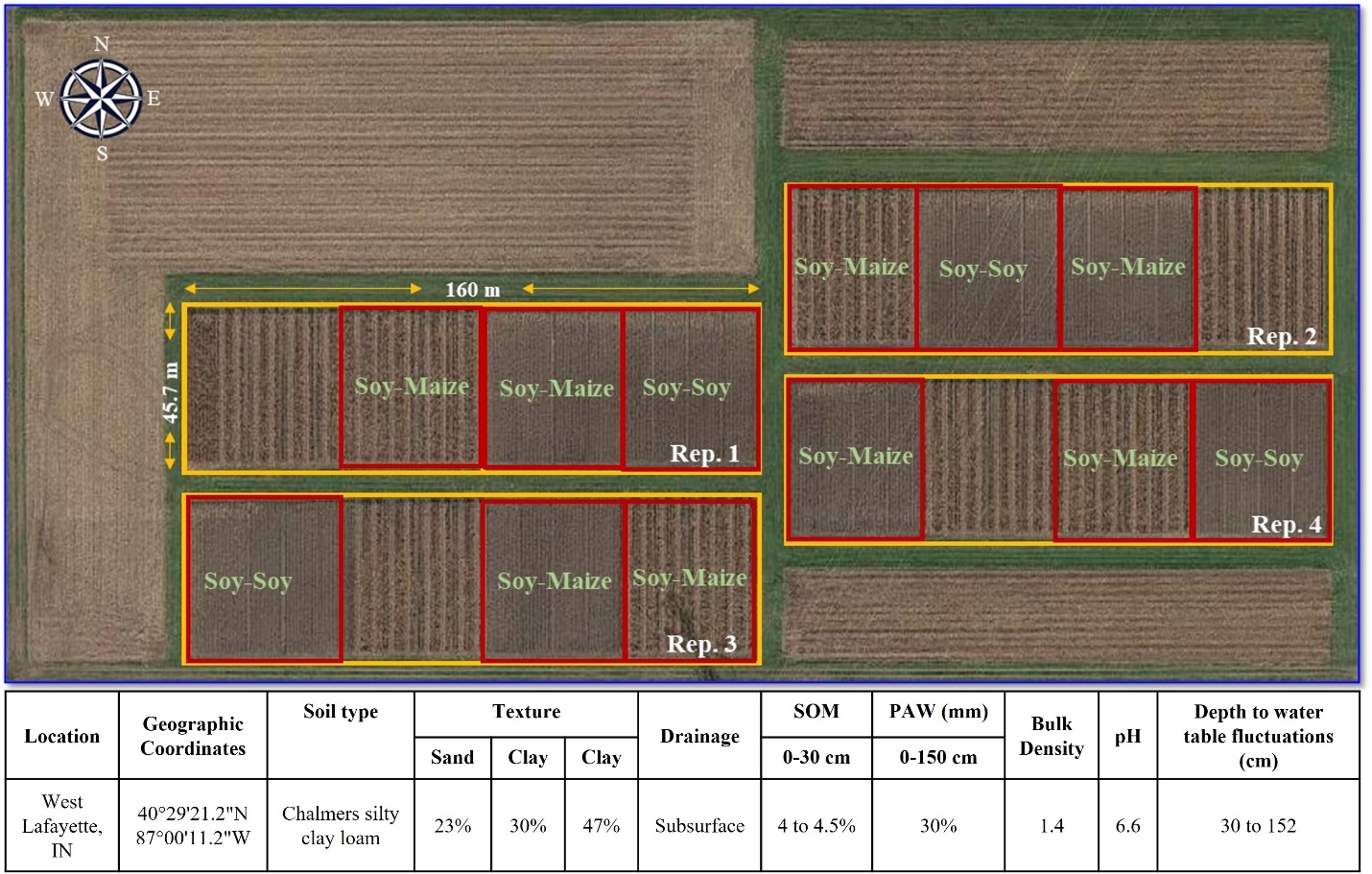
**

**Figure S1.** Experiment layout and soil characteristics. Continuous soybean and rotated with maize plots are depicted in this figure.

| **Year** | **Soybean Varieties** | **Planting Date** | **Harvest Date** |
| --- | --- | --- | --- |
| 1975 | Williams | 6-May | -- |
| 1976 | Williams | 10-May | 5-Oct |
| 1977 | Williams | 6-May | -- |
| 1978 | Williams | 19-May | 4-Oct |
| 1979 | Woodworth (Amsoy 71) | 17-May | 4-Oct |
| 1980 | Woodworth (Amsoy 71) | 15-May | 7-Oct |
| 1981 | Century | 28-May | 2-Oct |
| 1982 | Century | 11-May | 1-Oct |
| 1983 | Century | 12-May | 26-Sep |
| 1984 | Century | 5-May | 21-Sep |
| 1985 | Century | 16-May | 8-Oct |
| 1986 | Century | 28-May | 8-Oct |
| 1987 | Century | 7-May | 14-Sep |
| 1988 | Century 84 | 12-May | 25-Sep |
| 1989 | Century 84 | 12-May | 5-Oct |
| 1990 | Resnik | 21-May | 16-Oct |
| 1991 | Resnik | 16-May | 30-Sep |
| 1992 | Resnik | 8-May | 6-Oct |
| 1993 | Edison | 12-May | 4-Oct |
| 1994 | Edison | 17-May | 3-Oct |
| 1995 | Edison | 1-Jun | 30-Sep |
| 1996 | Ag Alumni 3351 | 21-Jun | 28-Oct |
| 1997 | Asgrow 3244 | 16-May | 3-Oct |
| 1998 | Asgrow 3244 | 18-May | 12-Oct |
| 1999 | Asgrow 3244 | 21-May | 14-Oct |
| 2000 | Pioneer 93B66 SCN resistant, Group (G) 3.6 | 24-May | 10-Oct |
| 2001 | Pioneer 93B66 SCN resistant, G 3.6 | 10-May | 5-Oct |
| 2002 | Pioneer 93B67 SCN resistant (G 3.6) | 29-May | 8-Oct |
| 2003 | Pioneer 93B67 (Roundup Ready, G 3.6) | 27-May | 9-Oct |
| 2004 | Pioneer 93B67 (Roundup Ready, G 3.6) | 4-Jun | 22-Oct |
| 2005 | Pioneer 93M80 (Roundup Ready, G 3.8) | 5-May | 5-Oct |
| 2006 | Pioneer 93M80 (Roundup Ready, G 3.8) | 31-May | 25-Oct |
| 2007 | Pioneer 93M82 (Roundup Ready, G 3.8) | 7-May | 9-Oct |
| 2008 | Pioneer 93M42 (Roundup Ready, G 3.4) | 28-May | 22-Oct |
| 2009 | Pioneer 93M61 (Roundup Ready, G 3.6) | 27-May | 22-Oct |
| 2010 | Pioneer 93M61 (Roundup Ready, G 3.6) | 30-May | 30-Sep |
| 2011 | Pioneer 93Y40 (Roundup Ready, G 3.4) | 12-May | 12-Oct |
| 2012 | Pioneer 93Y40 (Roundup Ready, G 3.4) | 25-April | 12-Oct |
| 2013 | Pioneer 93Y60 (Roundup Ready, G 3.6) | 17-May | 29-Oct |
| 2014 | Pioneer 93Y60 (Roundup Ready, G 3.6) | 23-May | 29-Oct |
| 2015 | Pioneer P35T97R2 (Roundup Ready, G 3.5) | 22-May | 6-Oct |
| 2016 | Pioneer P36T986R2 (Roundup Ready, G 3.6) | 23-May | 15-Oct |
| 2017 | Pioneer P34T07R2 (Roundup Ready, G 3.4) | 25-April | 21-Oct |
| 2018 | Pioneer P34T07R2 (Roundup Ready, G 3.4) | 1-May | 7-Oct |
| 2019 | Pioneer P34T07R2 Roundup Ready, G 3.4 | 11-Jun | 18-Oct |
| 2020 | Pioneer P34T21SE Roundup Ready, Xtend G 3.4 | 22-April | 7-Oct |
| 2021 | Pioneer P34T21SE Roundup Ready, Xtend G 3.4 | 21-May | 21-Oct |
| 2022 | Pioneer P37A18E Roundup Ready, Xtend G 3.7 | 14-May | 10-Oct |
| 2023 | Pioneer P37A18E Roundup Ready, Xtend G 3.7 | 10-May | 12-Oct |
| 2024 | Pioneer P37A18E Roundup Ready Xtend G 3.7 | 20-May | 11-Oct |

**TABLE S1.** Soybean varieties used, planting and harvest dates. G letters indicates maturity groups.

**
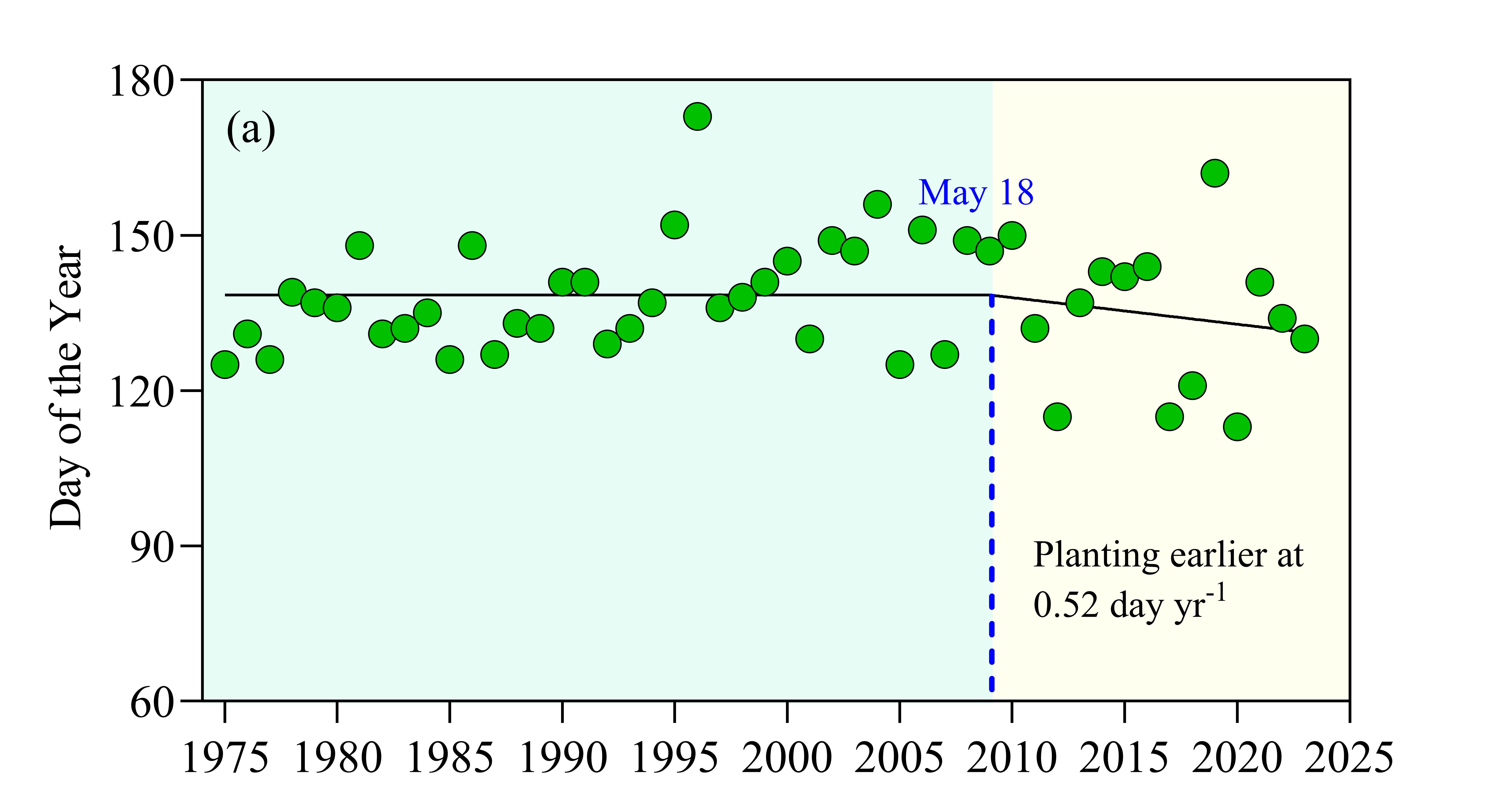
**

**FIGURE S2.** Planting date patterns over time. Green points show the day of year (DOY) Y-axis and actual day of planting for each year of experimentation on X-axis. Black line is the adjusted linear plateau model y = β0 + β1𝑥, if 𝑥 ≤ 𝑥𝑠, where: y is DOY, 𝑥 is the year, 𝑥𝑠 is the breakpoint, β0 is the intercept and β1 the slope. The line’s left portion indicates the experiment’s average planting date from 1975-2008 (May 18), and the blue dotted line indicates a transition point in planting day trends.

| ***Plant density (plants per m^2^)*** | | | |
| --- | --- | --- | --- |
| Source of Variation | DF Num / Den | F Value | *p-value* |
| Year (Y) | 44, 1072 | 1.08 | 0.3426 |
| Rotation (R) | 1, 1072 | 5.34 | 0.021 |
| Tillage (T) | 3, 1072 | 111.82 | <.0001 |
| R x T | 3, 1072 | 5.85 | 0.001 |
| ***Plant height at 4 weeks*** | | | |
| Source of Variation | DF Num / Den | F Value | *p-value* |
| Year (Y) | 43, 1056 | 367.15 | <.0001 |
| Rotation (R) | 1, 1056 | 19.22 | <.0001 |
| Tillage (T) | 3, 1056 | 67.49 | <.0001 |
| R x T | 3, 1056 | 6.36 | 0.001 |
| ***Plant height at 8 weeks*** | | | |
| Source of Variation | DF Num / Den | F Value | *p-value* |
| Year (Y) | 46, 1128 | 471.44 | <.0001 |
| Rotation (R) | 1, 1128 | 0.19 | 0.6615 |
| Tillage (T) | 3, 1128 | 119.92 | <.0001 |
| R x T | 3, 1128 | 21.2 | <.0001 |
| ***Height gain from 4 to 8 weeks*** | | | |
| Source of Variation | DF Num / Den | F Value | *p-value* |
| Year (Y) | 41, 1008 | 291.62 | <.0001 |
| Rotation (R) | 1, 1008 | 2.75 | 0.0976 |
| Tillage (T) | 3, 1008 | 47.05 | <.0001 |
| R x T | 3, 1008 | 10.57 | <.0001 |
| ***Seed yield*** | | | |
| Source of Variation | DF Num / Den | F Value | *p-value* |
| Year (Y) | 49, 1107 | 183.055.35 | <.0001 |
| Rotation (R) | 1, 1107 | 405.48 | <.0001 |
| Tillage (T) | 3, 1107 | 26.55 | <.0001 |
| R x T | 3, 1107 | 4.93 | 0.0021 |

**TABLE S2.** Statistical outcomes from the general linear mixed models for plant phenes (plant heights in cm), plant density (plants per m^2^) and seed yields (kg ha^-1^).


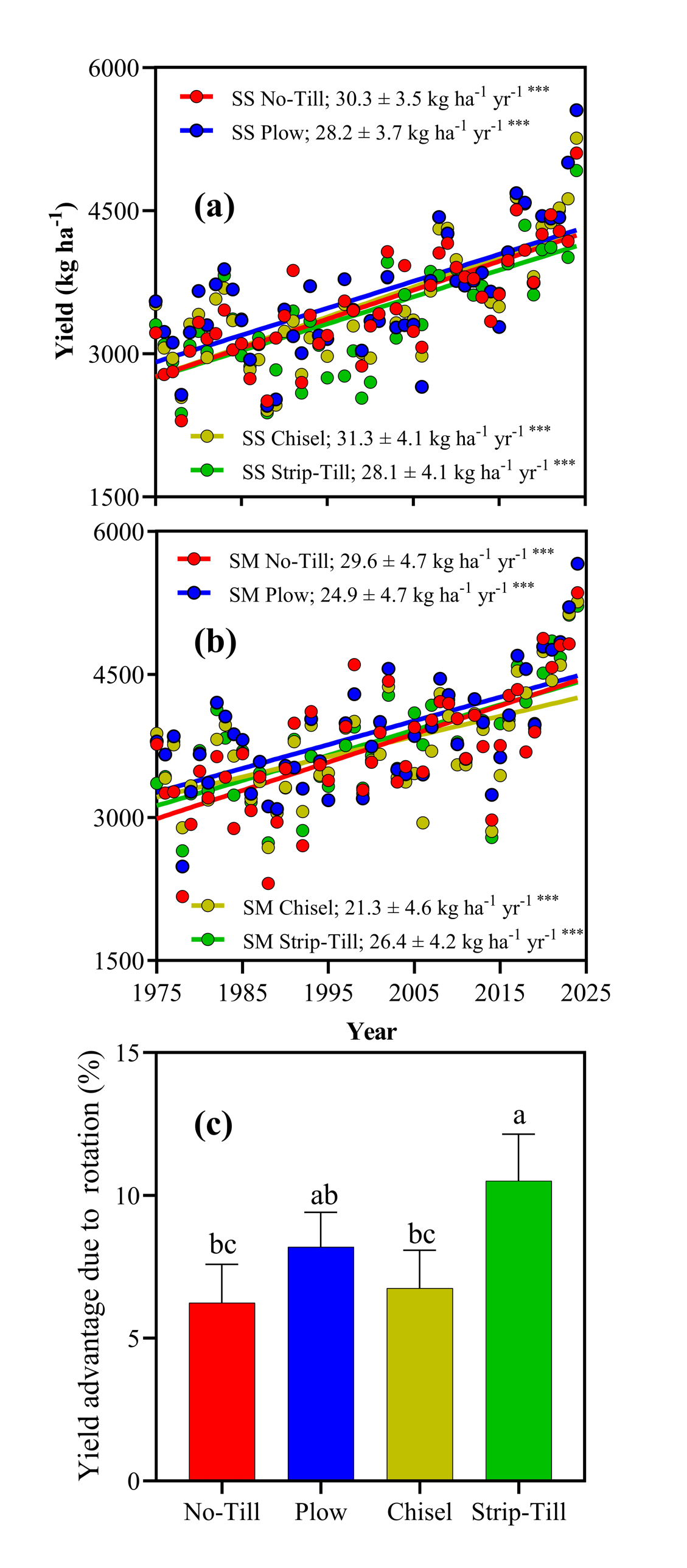


**FIGURE S3.** Soybean yield gain over time and yield advantage due to rotation in each tillage method. Annual soybean yield was regressed with years for continuous soybean (SS) in Fig. S3(a) and for soybean rotated with maize (SM) in Fig. S3(b). Tillage systems are denotated as follows: No-till in red, Moldboard Plow in blue, Chisel in gold, and Strip-Till in green. *P-values* for Figures S3(a) and S3(b) are reported as follows: *** = 0.001. Annual yield advantages were calculated for each tillage systems as a ratio of rotation relative to monocropping system and reported as a % in Fig. S3(c). Standard error bars were calculated across the 50-year time series to represent overall variability. The comparison of means was subjected to Tukeys’ HSD test. Different letters in Figure S3(c) indicate significant differences, with corresponding *P-values* set at 0.05.





**FIGURE S4**. Trend lines of the relationship between plant height, precipitation, and thermal time for the eight soybean production systems. Panels (a) and (b) shows the relationship trend-line between annual plant height average in (cm) and accumulated precipitation (mm) at 4 and 8 weeks after planting. Panels (c and d) displays the relationship trend-line between plant height and accumulated thermal time (^o^C day) at 4 and 8 weeks after planting. The coefficient of determination (R^2^) from linear regression analysis is reported with its respective significant levels. *P-values* are reported as follows: * = 0.05, ** = 0.01, *** = 0.001 and ns = not significant.





**FIGURE S5.** The relationships of yield, height gain per day at 4, and from 4 to 8 weeks versus management and weather variables. Low- and high-yielding conditions were determined by the 1^st^ and 4^th^ quartiles after ranking from low to high yields (n = 12), respectively. Coefficients of correlation (r) for final yield (a and b), plant height gain in cm per day from planting to 4 weeks (a and b), and plant height gains between 4 and 8 weeks after planting (a and b) with planting date, thermal unit accumulation (^o^C day) and cumulative precipitation (mm) for distinct time periods and yielding conditions for each tillage system under continuous soybean and soybean-maize rotation systems. Tillage systems are abbreviated as follows: NT = No-Till, MP = Moldboard Plow, CH = Chisel and ST = Strip-Till. Bars with positive values indicates that the slope on this regression was positive; negative bars reflect a negative regression value. Horizontal dotted lines in each panel indicate the levels of significance. When a bar reaches one of the horizontal dotted lines, it indicates that the relationship is significant at this level. *P-values* are reported as follows: 0.10, 0.05 and 0.001.


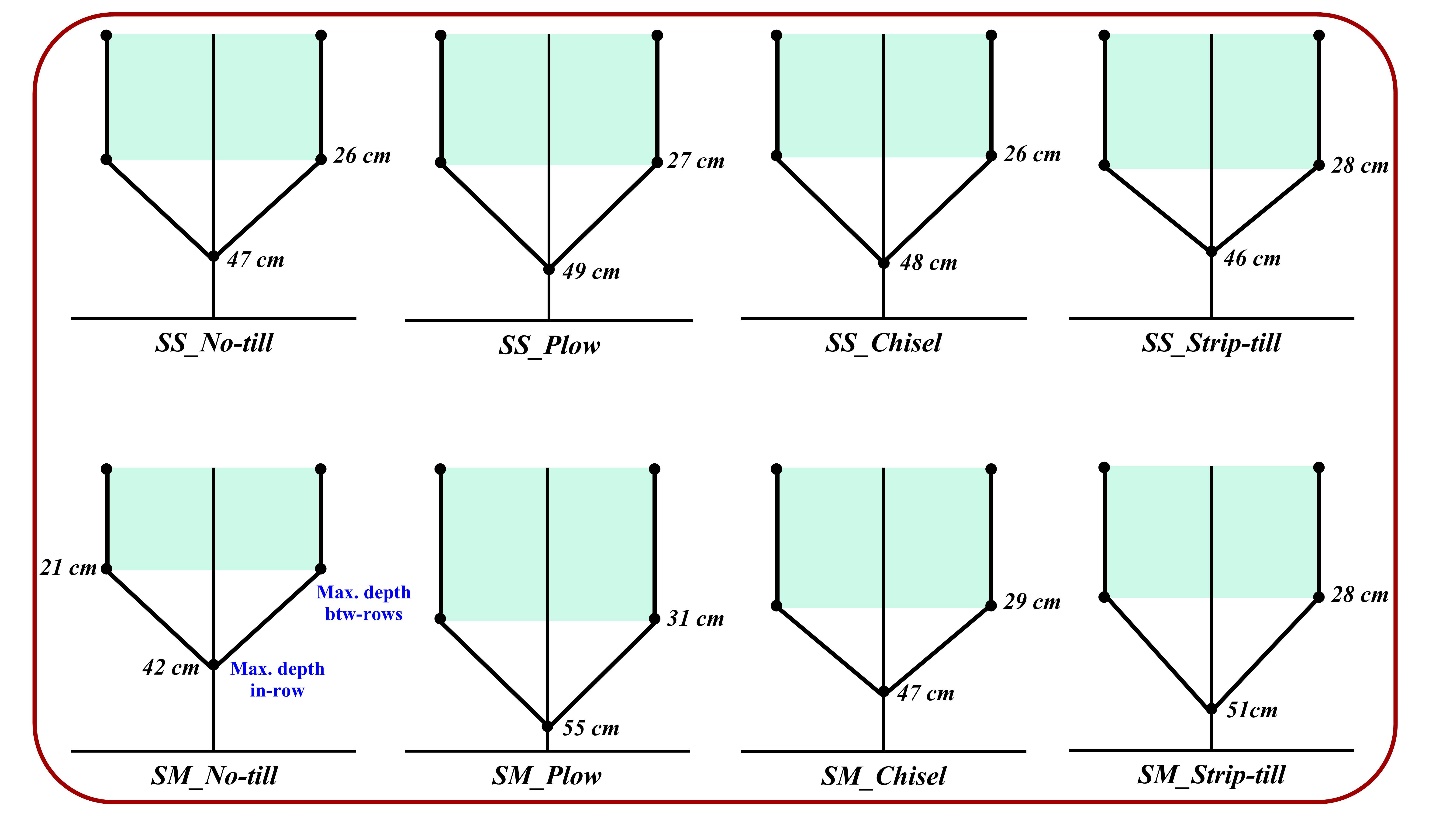


**FIGURE S6.** Maximum soybean root depth in-row and between two rows. Rooting depth (cm) measured at 4W after planting in monocropping and the soybean-maize rotation system across four tillage methods in year 2024.

| **Source of Variation** | | | Plant density (plants m^-2^) | Plant height at 4 weeks (cm) | Plant height at 8 weeks (cm) | Height gain from 4 to 8 weeks (cm) | Seed yield (kg ha^-1^) |
| --- | --- | --- | --- | --- | --- | --- | --- |
| **Rotation (R)** | Soybean-Maize | | 42.5a | 13.3a | 50.4a | 37.1a | 3638a |
|  | Continuous Soybean | | 42.7a | 13.1a | 48.2b | 35.1b | 3235b |
| **Tillage (T)** | Plow | | 45.3b | 12.9b | 50.0a | 37.1a | 3515ab |
|  | Chisel | | 45.3b | 13.1b | 49.5a | 36.4ab | 3404bc |
|  | Ridge-Till | | 31.6c | 14.2a | 50.3a | 36.1ab | 3299c |
|  | No-Till | | 48.2a | 12.6b | 47.2b | 34.6b | 3529a |
| **R x T** | Soybean-Maize | Plow | 45.3a | 13.2ab | 51.7ab | 38.5a | 3713a |
|  |  | Chisel | 45.1a | 13.1ab | 50.1abc | 37.0abc | 3578a |
|  |  | Ridge-Till | 31.5b | 14.3a | 52.2a | 37.9ab | 3569a |
|  |  | No-Till | 48.1a | 12.7b | 47.6cd | 34.9bc | 3691a |
|  | Continuous Soybean | Plow | 45.2a | 12.6b | 48.4cd | 35.8abc | 3316b |
|  |  | Chisel | 45.5a | 13.1ab | 49.0bcd | 35.9abc | 3230b |
|  |  | Ridge-Till | 37.7b | 14.1ab | 48.5cd | 34.4c | 3028c |
|  |  | No-Till | 48.3a | 12.6b | 46.9d | 34.3c | 3367b |

**TABLE S3.** Agronomic, morphophysiological traits and seed yields measured in each tillage and rotation combination from 1995 to 2004 when soybeans were drill-seeded in 19-cm row widths for Plow, Chisel and No-Till systems while Ridge-Till was seeded in 76-cm row widths, as had been the standard practice for all tillage systems before 1995 and after 2004. Plant density was assessed at 4 weeks after planting and reported in plants per square meter. Plant height data was collected at 4 and 8 weeks after planting and reported in cm. Yields averages for each tillage and rotation system are reported in kg per hectare at 13% moisture. Tukeys’ HSD test was implemented for pairwise comparisons of means with α set at 0.05. Different letters denotate significant differences between means.

| **Timing** | **Weather variables** | **Low productivity 1^st^ quartile** | **High productivity 4^th^ quartile** |
| --- | --- | --- | --- |
| Sowing to 4 Weeks | Cumulative Precipitation (mm) | 76.1 ± 55 | 108.1 ± 56 |
|  | Cumulative TT (^o^C day) | 281.8 ± 41.3 | 280.3 ± 76.5 |
|  | Min. Temperature (^o^C) | 13.0 ± 1.9 | 13.2 ± 3.2 |
|  | Max. Temperature (^o^C) | 26.2 ± 1.9 | 25.9 ± 3.5 |
|  | Mean Temperature (^o^C) | 19.6 ± 1.7 | 19.6 ± 3.3 |
| From 4 to 8 Weeks | Cumulative Precipitation (mm) | 89.4 ± 61.2 | 97.7 ± 51.7 |
|  | Cumulative TT (^o^C day) | 346.5 ± 39.8 | 368.0 ± 35.8 |
|  | Min. Temperature (^o^C) | 16.0 ± 1.8 | 17.2 ± 1.6 |
|  | Max. Temperature (^o^C) | 28.6 ± 1.7 | 29.0 ± 1.3 |
|  | Mean Temperature (^o^C) | 22.3 ± 1.5 | 23.1 ± 1.3 |
| Sowing to 8 weeks | Cumulative Precipitation (mm) | 165.4 ± 75.4 | 205.8 ± 6.3 |
|  | Cumulative (^o^C day) | 628.3 ± 71.0 | 648.3 ± 94.3 |
|  | Min. Temperature (^o^C) | 14.5 ± 1.8 | 15.2 ± 2.0 |
|  | Max. Temperature (^o^C) | 27.4 ± 1.6 | 27.4 ± 2.0 |
|  | Mean Temperature (^o^C) | 20.9 ± 1.4 | 21.3 ± 1.9 |

**TABLE S4.** Weather variables measured across different time periods. Weather data include the cumulative precipitation (mm) and thermal time (^o^C day), the mean of both minimum and maximum temperature (^o^C), alongside the mean of these temperatures. Corresponding standard deviations (n = 12) are provided for all weather values.
